# Venous Thromboembolism in Neonates, Children, and Adolescents: A Comprehensive Narrative Review of Risk Factors, Diagnosis, Treatment, and Prevention

**DOI:** 10.3390/medicina62091712

**Published:** 2026-09-06

**Authors:** Marko Bašković, Jana Buzuk, Bianka Dujić, Danijela Jurić, Kristina Jurković, Karla Pehar, Sara Vuković, Katarina Čavka, Miroslav Gjurašin, Dubravko Habek, Davor Bojić, Darko Antičević, Katarina Lohman Vuga, Ivan Milas

**Affiliations:** 1 School of Medicine, Catholic University of Croatia, Ilica 242, 10000 Zagreb, Croatia; 2 Department of Pediatric Surgery, Children’s Hospital Zagreb, Ulica Vjekoslava Klaića 16, 10000 Zagreb, Croatia; 3 School of Medicine, University of Zagreb, Šalata 3, 10000 Zagreb, Croatia; 4 Scientific Centre of Excellence for Reproductive and Regenerative Medicine, School of Medicine, University of Zagreb, Šalata 3, 10000 Zagreb, Croatia; 5 Croatian Academy of Medical Sciences, Kaptol 15, 10000 Zagreb, Croatia; 6 Referral Center for Pediatric Traumatology, Children’s Hospital Zagreb, Ulica Vjekoslava Klaića 16, 10000 Zagreb, Croatia; 7 School of Medicine, University of Split, Šoltanska Ulica 2a, 21000 Split, Croatia; 8 Department of Pediatric Neurosurgery, Children’s Hospital Zagreb, Ulica Vjekoslava Klaića 16, 10000 Zagreb, Croatia; 9 Department of Obstetrics and Gynecology, Clinical Hospital Merkur, Zajčeva Ulica 19, 10000 Zagreb, Croatia; 10 Department of Pediatric Orthopedics, Children’s Hospital Zagreb, Ulica Vjekoslava Klaića 16, 10000 Zagreb, Croatia; 11 Faculty of Medicine, Josip Juraj Strossmayer University of Osijek, Josipa Huttlera 4, 31000 Osijek, Croatia; 12 St. Catherine Specialty Hospital, Ulica Kneza Branimira 71E, 10000 Zagreb, Croatia; 13 Faculty of Dental Medicine and Health, Josip Juraj Strossmayer University of Osijek, Crkvena 21, 31000 Osijek, Croatia; 14 Special Hospital for Medical Rehabilitation Varaždinske Toplice, Trg Svetog Martina 1, 42223 Varaždinske Toplice, Croatia; 15 Department of Surgical Oncology, University Hospital for Tumors, University Hospital Centre Sestre Milosrdnice, Ilica 197, 10000 Zagreb, Croatia

**Keywords:** venous thromboembolism, children, neonatal thrombosis, central venous catheter, deep vein thrombosis, pulmonary embolism, cerebral sinovenous thrombosis, anticoagulation, thromboprophylaxis, direct oral anticoagulants

## Abstract

Venous thromboembolism (VTE) was once considered rare in the young, but it has become an increasingly important complication of contemporary pediatric care, driven by the improved survival of children with complex chronic illness and by the expanding use of central venous catheters. This narrative review synthesizes current evidence on VTE across the entire pediatric age range, from the critically ill neonate to the injured adolescent. We first examine noncerebral VTE in children beyond the newborn period, describing an incidence that is far lower than in adults yet rising among hospitalized patients, the multifactorial risk factors dominated by central venous catheters, and the age-dependent protection conferred by developmental hemostasis. We outline a diagnostic approach centered on compression ultrasonography and computed tomography pulmonary angiography, and a treatment paradigm that increasingly favors direct oral anticoagulants and shorter, six-week courses for low-risk provoked events. Dedicated sections address the distinct biology, presentation, and management of neonatal thrombosis, including renal vein thrombosis, portal vein thrombosis, and purpura fulminans, for which low-molecular-weight heparin is preferred and warfarin is generally avoided. We review the heightened, malignancy-specific risk of cancer-associated thrombosis, the difficulty of anticoagulating the thrombocytopenic child, and the consistent evidence against routine primary thromboprophylaxis. Cerebral sinovenous thrombosis is considered in depth, emphasizing its age-dependent triggers, the central role of magnetic resonance venography, and the safety of anticoagulation. Finally, we summarize the comparatively low but age-graded risk of VTE after major pediatric trauma and the puberty-based approach to prophylaxis. Throughout, we highlight the continued reliance on extrapolated adult data, the emergence of pediatric randomized trials and multicenter registries, and the unmet need for prospectively validated risk-prediction tools. The review offers clinicians an integrated, contemporary framework for recognizing, diagnosing, treating, and preventing thrombosis from the neonate to the adolescent.

## 1. Introduction

Venous thromboembolism (VTE) was long regarded as a disease of adults and an uncommon, easily overlooked event in childhood. Over the past two decades, however, it has emerged as an increasingly important complication of pediatric care, now recognized as a distinct clinical entity whose epidemiology, pathophysiology, and management differ substantially from those in adults [1,2,3,4,5,6,7,8].

This rising clinical importance is concentrated in hospital-based cohorts and is driven less by any change in the intrinsic thrombotic propensity of children than by the improved survival of neonates and children with complex illness and by the expanding use of central venous catheters [3,4,5,8]. In most affected children, thrombosis is multifactorial, arising when one or more acquired triggers are superimposed on a predisposed host, and the central venous catheter is the single most important contributor [1,7]. However, children remain far less prone to thrombosis than adults, a relative protection that reflects both their limited exposure to adult-pattern risks and the physiologic properties of developmental hemostasis [2]. These distinctive features, together with the historical scarcity of high-quality pediatric data and the resulting reliance on extrapolation from adult practice, are examined in detail in the sections that follow.

Against this background, a consolidated and contemporary synthesis is needed to guide clinicians who care for children across the full pediatric age range. The objective of this narrative review is to provide an integrated, up-to-date overview of venous thromboembolism in neonates, children, and adolescents, encompassing its epidemiology and risk factors, the pathophysiologic role of developmental hemostasis, and current approaches to diagnosis, treatment, and prevention. The review also addresses the particular challenges posed by high-risk populations, including neonates, children with cancer, those with cerebral sinovenous thrombosis, and the severely injured, with the aim of offering clinicians a practical framework for recognizing, diagnosing, treating, and preventing thrombosis from the neonate to the adolescent. It is organized primarily according to patient age and clinical setting rather than anatomical site, with successive sections devoted to children and adolescents, neonates, children with cancer, cerebral sinovenous thrombosis, the severely injured trauma patient, and other special populations, and with anatomical location treated only as a secondary level of detail within the diagnostic and treatment subsections, where the clinical approach genuinely differs from one site to another.

## 2. Venous Thrombosis and Thromboembolism in Children and Adolescents

### 2.1. Incidence and Epidemiology

In the general pediatric population, VTE is uncommon, with population-based estimates of the annual incidence ranging from roughly 0.07 to 0.49 cases per 10,000 children [1,7]. The burden is heavily concentrated among hospitalized children, in whom reported rates are far higher, on the order of 0.2 to 1 percent of admissions, and have climbed steadily over the past two decades [3,4,8]. Analyses of large administrative datasets have documented this rise, with the annual rate of VTE among hospitalized children increasing several-fold between the early 2000s and the late 2010s; whether this trend reflects a true increase in disease or improved ascertainment through more frequent and more sensitive imaging remains debated [4,9]. Pulmonary embolism (PE), historically regarded as exceptional in children, accounts for only a minority of pediatric VTE episodes but has shown a parallel increase in diagnosis over the same period [9].

Rates are consistently highest in children cared for at tertiary referral centers and in those with serious underlying disease, particularly malignancy and congenital or acquired heart disease [3,8]. One analysis found an approximately fivefold higher incidence of VTE among children admitted to tertiary children’s hospitals than among those managed at community hospitals, a difference attributable largely to the complexity of care, including central venous access, major surgery, and critical illness, rather than to any intrinsic property of the institutions themselves [8]. Across registries, the great majority of pediatric VTE events arise in the inpatient setting, and nearly all affected children carry at least one identifiable predisposing factor [1,10].

The age distribution of childhood VTE is characteristically bimodal, with one peak in infancy and a second during adolescence [1,2]. The infant peak is driven largely by critically ill neonates and young infants in whom indwelling catheters are common, whereas the adolescent peak reflects the emergence of adult-type risk factors such as obesity, estrogen-containing contraceptives, and pregnancy [7,11]. In a nationwide Danish registry, most children with noncerebral thromboembolism had at least one underlying condition or trigger, approximately one-third were found to harbor an inherited thrombophilic trait, and estrogen-containing contraceptive use was a dominant risk factor among adolescent girls [7].

### 2.2. Why Children Are Relatively Protected from Venous Thromboembolism

The markedly lower frequency of VTE in children than in adults cannot be explained by reduced exposure to risk factors alone. It also reflects fundamental, age-dependent properties of the developing hemostatic system [2,12]. Several lines of protection operate throughout childhood. Children rarely have the chronic vascular pathology, such as diabetes, dyslipidemia, atherosclerosis, and hypertension, that injures the endothelium and predisposes adults to thrombosis [1,2]. They are also far less often exposed to acquired prothrombotic states, including oral contraceptive and hormone-replacement use, pregnancy and the puerperium, smoking, malignancy, and major orthopedic surgery [1,11].

Beyond these epidemiologic differences, the concept of developmental hemostasis captures quantitative and qualitative differences in coagulation between children and adults that are physiologic rather than pathologic [12]. Compared with adults, children have lower circulating concentrations of most of the vitamin K-dependent and contact coagulation factors and a correspondingly reduced capacity to generate thrombin [2,12]. At the same time, the capacity to inhibit thrombin is enhanced, owing in part to higher plasma concentrations of the broad-spectrum protease inhibitor α_2_-macroglobulin [12]. The net effect is a more tightly regulated, comparatively thromboprotective hemostatic balance, which helps to explain why even children who inherit a thrombophilic defect frequently remain free of thrombosis unless an additional acquired trigger is superimposed [11,12].

### 2.3. Risk Factors

The pathophysiology of pediatric VTE conforms to the triad described by Virchow more than a century and a half ago: endothelial injury, venous stasis, and a hypercoagulable state [6,11]. In most children, thrombosis is multifactorial, developing when two or more of these elements coincide, typically when an acquired clinical trigger is superimposed on a critically or chronically ill child [1,11]. By a wide margin, the single most important and most prevalent risk factor in childhood is the presence of a central venous catheter (CVC); other major contributors include inherited thrombophilia, serious infection, trauma, immobility, malignancy, congenital heart disease, and chronic inflammatory disorders (Table 1) [1,2,13].

#### 2.3.1. Central Venous Access Devices

Central venous catheters (CVCs) are implicated in roughly one-third to two-thirds of all venous thromboses in children, which makes them the single leading cause of pediatric VTE [1,5]. These devices are indispensable to contemporary care, providing dependable access for parenteral nutrition, chemotherapy, prolonged antimicrobial therapy, vasoactive infusions, and repeated blood sampling in critically and chronically ill children [2,5]. Their thrombogenicity is intrinsic: an indwelling catheter presents a continuous foreign surface to flowing blood, mechanically injures the vessel wall during insertion and dwell, and disturbs normal laminar flow, thereby engaging all three arms of Virchow’s triad at once [5].

Several device- and patient-related variables modulate the risk of catheter-associated thrombosis:

Catheter type. In most studies, peripherally inserted central catheters (PICCs) carry a higher thrombotic risk than tunneled or non-tunneled CVCs, with reported event rates of roughly 1–9% for PICCs versus about 0.5–3% for other devices; a minority of studies, however, report comparable or even lower rates for PICCs [14,15,16,17,18].

Insertion site. Thrombosis may involve the upper venous system (jugular or subclavian lines) or the iliofemoral system and lower extremities (femoral lines); some data indicate a higher risk with upper-extremity catheters, whereas other series find similar rates regardless of site [14,17].

Catheter caliber. The risk increases as the diameter of the catheter rises relative to that of the cannulated vein [5,17].

Number of lumens. Multi-lumen catheters appear more thrombogenic than single-lumen devices [5,17].

Coexisting prothrombotic conditions. Underlying malignancy, trauma, infection, or inherited thrombophilia further amplifies the thrombotic risk of an indwelling line [5,19].

Catheter-to-vein diameter ratio. Beyond the absolute caliber of the device, the ratio between the external diameter of the catheter and the diameter of the cannulated vein is an important determinant of blood flow around the line, and a larger ratio favors stasis and thrombus formation. International pediatric recommendations advise that the catheter should occupy no more than roughly one third of the vein diameter, and prospective pediatric data link both a higher catheter-to-vein diameter ratio and a higher catheter-to-vein area ratio to an increased thrombotic risk [5,20,21].

Catheter material. The composition and surface characteristics of the device also modulate its thrombogenicity, and softer, more biocompatible materials such as silicone and polyurethane are generally preferred over stiffer, more thrombogenic plastics [5].

Catheter-related infection. Catheter-associated thrombosis and central line-associated bloodstream infection are closely linked in a bidirectional fashion, because the fibrin sheath that forms around the catheter favors microbial adhesion, while the inflammatory response to bloodstream infection in turn promotes further thrombus growth [5,20].

Number of punctures and vessel trauma. Repeated or difficult cannulation attempts and the resulting endothelial injury add to the thrombotic risk, and catheters placed by anatomical landmark or open surgical techniques have been associated with more thrombosis than those placed under real-time ultrasound guidance [5,22,23].

#### 2.3.2. Inherited Thrombophilia

Although inherited thrombophilia is firmly established as a risk factor for VTE in adults, its contribution to thrombosis during childhood is comparatively modest and continues to be debated [11,19]. Because the developing hemostatic system is intrinsically thromboprotective, many children who carry a thrombophilic defect never experience a thrombotic event unless an acquired trigger is superimposed [11,12]. The abnormalities with the best-established causal link to pediatric VTE are the gain-of-function factor V Leiden and prothrombin G20210A mutations and deficiencies of the natural anticoagulants antithrombin, protein C, and protein S [11].

Reported estimates of the prevalence of inherited thrombophilia among children with VTE vary widely, from roughly 10% to 60%, reflecting differences in the populations studied, the proportion of provoked versus unprovoked events, and the breadth of the laboratory panel applied [11,24]. The diagnostic yield is highest in children with spontaneous (unprovoked) thrombosis and lowest in those whose event is clearly attributable to a central venous catheter; consistent with this, meta-analytic data do not support a strong independent association between thrombophilia and catheter-related thrombosis in children [11,19]. These observations underpin a selective rather than universal approach to thrombophilia testing [11].

#### 2.3.3. Other Clinical Risk Factors

A wide range of additional acquired conditions increases thrombotic risk in children, most of them by establishing one or more components of Virchow’s triad: a protein-losing or inflammatory state, vascular disruption, or venous stasis (Table 1) [1,13].

Age. The incidence of pediatric VTE is bimodal, with an infant peak related to indwelling catheters and an adolescent peak reflecting adult-pattern risk factors, as detailed in Section 2.1 [1,7].

Severe infection and sepsis. Serious systemic bacterial infection predisposes to VTE and is classically linked with meningococcal disease, although any pathogen may be responsible [1,13]. Viral infections can also provoke thrombosis, as is well documented for SARS-CoV-2 infection and the associated multisystem inflammatory syndrome in children [25]. Pulmonary embolism should be suspected in a child with pneumonia whose hypoxemia persists or worsens despite appropriate antimicrobial therapy, a presentation increasingly recognized with *Mycoplasma pneumoniae* infection [26].

Malignancy. VTE may complicate any childhood cancer, usually during treatment rather than at diagnosis, with risk modulated by tumor biology, vascular compression, and the therapeutic regimen. Thrombosis is especially frequent in children receiving asparaginase for acute lymphoblastic leukemia, whereas it is comparatively uncommon in those with primary brain tumors [2].

Congenital heart disease. Children with congenital heart disease are at heightened risk, particularly those with single-ventricle (Fontan) physiology or surgically implanted conduits and prosthetic valves, in whom low-flow states and artificial surfaces favor thrombus formation [1,13].

Trauma and surgery. Major trauma raises thrombotic risk, though far less than in injured adults; reported rates of VTE in pediatric trauma populations range from roughly 0.03% to more than 1%, with the highest rates among children sustaining major vascular injury or requiring orthopedic surgery [27,28,29]. The risk rises steeply with advancing age toward adolescence [28].

Estrogen-containing contraceptives. Use of estrogen-containing contraceptives is an important and potentially modifiable risk factor for VTE in adolescent girls, especially when combined with other prothrombotic conditions [7,11].

Nephrotic syndrome and protein-losing states. Urinary loss of the natural anticoagulants in nephrotic syndrome produces an acquired hypercoagulable state that predisposes to venous thrombosis, including thrombosis of the renal veins [30].

Inflammatory bowel disease. Children with inflammatory bowel disease carry an approximately two- to threefold higher risk of VTE than unaffected peers, and the risk increases markedly during disease flares [31,32,33].

Autoimmune disease. Systemic lupus erythematosus and the antiphospholipid syndrome are associated with an elevated thrombotic risk in children, mediated by antiphospholipid antibodies and chronic systemic inflammation [1].

Vascular anomalies. Certain anatomic and vascular abnormalities predispose to localized thrombosis, including May–Thurner syndrome, thoracic outlet (Paget–Schroetter) syndrome, and inferior vena cava atresia [2].

Other factors in hospitalized children. In hospitalized children, prolonged immobility, critical illness, and the duration of mechanical ventilation are further considerations that inform decisions about thromboprophylaxis [13,34,35].

### 2.4. Clinical Manifestations

The clinical expression of VTE in children is highly variable and depends chiefly on the anatomic location of the thrombus and the degree to which it occludes the affected vessel [2]. The spectrum extends from a complete absence of symptoms to limb-threatening or life-threatening presentations.

#### 2.4.1. Catheter-Associated Thrombosis

Most catheter-associated thromboses are clinically silent and are discovered only when imaging is undertaken for another reason [2,5]. When manifestations do arise, they reflect either impaired catheter function or venous obstruction and may include repeated loss of catheter patency, catheter-associated bloodstream infection, swelling and discoloration of the ipsilateral limb, facial swelling, and the distended superficial collateral veins of the superior vena cava syndrome [2,5]. Less frequently, a catheter-associated thrombus gives rise to pulmonary embolism, to paradoxical embolism and stroke in children with a right-to-left intracardiac shunt, or to chylothorax [2,5].

#### 2.4.2. Deep Vein Thrombosis

Outside the setting of central venous catheters, deep vein thrombosis most often involves the lower extremities, particularly the iliac, femoral, and popliteal veins, and presents with unilateral pain, swelling, and reddish or purple discoloration of the leg, sometimes accompanied by a measurable difference in calf circumference between the two limbs. Homans’ sign is unreliable in children and should not be relied upon to confirm or exclude the diagnosis. Non-catheter-related upper-extremity deep vein thrombosis is rare. When present, it produces unilateral swelling, discoloration, and discomfort of the arm, with neck or shoulder pain and facial swelling if the thrombus extends into the superior vena cava. Effort-related (primary) upper-extremity thrombosis, the Paget–Schroetter syndrome, is an uncommon entity that typically affects otherwise healthy young people, most often males [2].

#### 2.4.3. Pulmonary Embolism

Pulmonary embolism is uncommon in children but must be considered whenever a critically ill child deteriorates unexpectedly. It may present with pleuritic chest pain, dyspnea, tachypnea, cough, tachycardia, hypoxemia, or sudden cardiovascular collapse, and concurrent signs of deep vein thrombosis may be evident. In many children, however, especially the very young, the presentation is nonspecific and is readily mistaken for the underlying illness [36]. The recognized incidence of pediatric pulmonary embolism has increased since the early 2000s, paralleling the broader rise in childhood VTE [9]. Commonly cited risk factors include central venous catheters, trauma, immobility, recent surgery, estrogen-containing contraceptives, inflammatory conditions, malignancy, heart disease, dehydration, and obesity [36,37].

Most pediatric pulmonary emboli are nonmassive and hemodynamically inconsequential. In a cohort of 170 children with pulmonary embolism, 71% of events were nonmassive and 29% were massive or submassive, and all 11 deaths (6%) occurred among children with massive or submassive disease, who were also younger and more likely to have a central venous catheter or underlying cardiac condition [38]. Echocardiographic evidence of right ventricular strain carries adverse prognostic significance in this setting [39].

#### 2.4.4. Other Sites of Venous Thrombosis

Renal vein thrombosis in children most often complicates nephrotic syndrome or renal transplantation. Its onset is frequently insidious and may first become apparent through extension into the inferior vena cava or through embolization, although hematuria, proteinuria, oliguria, and a palpable flank mass can occur [30]. In the neonatal period, renal vein thrombosis is the most common non-catheter-related VTE [40]. Portal vein thrombosis may follow liver transplantation, intra-abdominal infection, splenectomy, or chemotherapy, or may complicate sickle cell disease or the antiphospholipid syndrome. It can present acutely with abdominal pain or remain occult until the sequelae of chronic portal hypertension, splenomegaly, and bleeding from esophageal varices supervene [30]. In neonates, portal vein thrombosis is most often related to umbilical venous catheterization and sepsis [41]. Cerebral venous thrombosis is addressed separately in this review.

### 2.5. Diagnostic Evaluation

The diagnosis of venous thrombosis in children rests on imaging, and the choice of modality is guided chiefly by the suspected location of the thrombus. Compression (duplex) ultrasonography is the preferred initial study in most circumstances because it is non-invasive, widely available, requires neither sedation nor ionizing radiation, and demonstrates the diagnostic hallmark of a non-compressible vein, with or without directly visible intraluminal thrombus [2,24]. Although pediatric validation data are limited, ultrasonography is considered to have acceptable sensitivity and specificity for proximal venous thrombosis by extrapolation from adult studies [2]. Alternative modalities, contrast venography, magnetic resonance venography (MRV), and computed tomography (CT) venography are reserved for situations in which ultrasonography is anatomically limited or inconclusive [2,24].

#### 2.5.1. Evaluation According to Anatomic Site

Catheter-associated thrombosis. Children with signs of large-vessel catheter-associated thrombosis should undergo duplex ultrasonography; when a purely mechanical catheter malfunction is suspected, instillation of contrast through the lumen under fluoroscopy can reveal thrombus at the catheter tip [24].

Lower-extremity deep vein thrombosis. Compression ultrasonography is the first-line investigation; if it is normal but clinical suspicion remains high, it may be repeated after about one week, and MRV (or CT where MRV is unavailable) is appropriate when proximal iliofemoral extension is suspected [24].

Upper-extremity and central thoracic veins. The peripheral upper-extremity, axillary, subclavian, and internal jugular veins are assessed initially with ultrasonography, but this technique is relatively insensitive for thrombi in the central intrathoracic veins because the clavicle and thoracic cage impede both visualization and venous compression; MRV is therefore preferred for definitive assessment of the central veins because it avoids radiation, with CT or contrast venography as alternatives [2,24].

#### 2.5.2. Suspected Pulmonary Embolism

Computed tomography pulmonary angiography (CTPA) is the imaging modality of choice for the diagnosis of pulmonary embolism in children [42,43]. Clinical prediction tools validated in adults, such as the Wells score and D-dimer testing, perform poorly in the pediatric setting and do not reliably distinguish children with radiographically confirmed PE from those without it [36]. Ventilation-perfusion scintigraphy and magnetic resonance pulmonary angiography avoid ionizing radiation but are more time-consuming and frequently require sedation in young children, so their use is generally confined to situations in which CTPA is contraindicated, unavailable, or inconclusive [43]. Echocardiography is insensitive and nonspecific for the diagnosis itself but may yield supportive and prognostic information, notably evidence of right ventricular strain [39].

### 2.6. Thrombophilia Testing

Once VTE has been confirmed, testing for inherited or acquired thrombophilia may be considered, but its value depends heavily on the clinical context [11]. Because catheter-associated thrombosis is overwhelmingly driven by the device itself, routine thrombophilia testing is generally not warranted after a first, clearly catheter-provoked event [11,19]. A selective strategy, reserving comprehensive testing for children with spontaneous or recurrent thrombosis, a strong family history, or thrombosis at an unusual site, maximizes the chance that a positive result will alter management while avoiding the cost, the interpretive difficulties created by age-dependent reference ranges, and the psychological burden of indiscriminate screening [11,12].

### 2.7. Differential Diagnosis

The differential diagnosis of VTE depends on the site of suspected thrombosis [2]. Conditions that mimic upper- or lower-extremity deep vein thrombosis include those that produce limb swelling, erythema, and tenderness, such as cellulitis, a ruptured popliteal (Baker’s) cyst, musculoskeletal injury, lymphangitis or lymphatic obstruction, and superficial thrombophlebitis, and although the history and examination usually narrow the possibilities, ultrasonography is ultimately required for confirmation [2]. Suspected pulmonary embolism must be distinguished from the many other causes of pediatric chest pain, dyspnea, and hypoxemia, including pneumonia, pneumothorax, asthma, pericarditis, and musculoskeletal chest-wall pain [36].

### 2.8. Goals and General Principles of Treatment

The aims of treating VTE in children are to prevent extension and embolization of the thrombus, promote its resolution, prevent recurrence, and minimize long-term sequelae such as the post-thrombotic syndrome (PTS) [44]. Anticoagulation is the cornerstone of therapy; the agents in routine pediatric use are low-molecular-weight heparin (LMWH), unfractionated heparin (UFH), the direct oral anticoagulants (DOACs), and vitamin K antagonists (VKAs, principally warfarin) [44]. Thrombolysis is reserved for the uncommon situation in which a thrombus threatens life, limb, or a vital organ [44]. Because high-quality pediatric evidence remains limited, management still leans heavily on extrapolation from adult data, expert consensus, and accumulated clinical experience, and is best anchored to the 2024 ASH/ISTH treatment recommendations; involvement of a pediatric hematologist is advisable in most cases [44].

### 2.9. Indications for and Duration of Anticoagulation

#### 2.9.1. Catheter Occlusion and Superficial Vein Thrombosis

Superficial thromboses associated with peripheral intravenous catheters are common in hospitalized children but are usually asymptomatic and call only for removal of the catheter and local observation rather than anticoagulation. When a central venous catheter is occluded without a demonstrable thrombus, instillation of recombinant tissue plasminogen activator (alteplase) into the lumen can restore patency. A volume equal to the internal volume of the lumen is instilled (up to 1 mL of a 0.5 mg/mL solution in children weighing 10 kg or less and up to 2 mL of a 1 mg/mL solution in larger children), left to dwell for one to two hours, and then aspirated, with a second course given if the first fails. Persistent occlusion thereafter should prompt exclusion of catheter malposition or kinking and imaging to look for catheter-associated thrombosis [44].

#### 2.9.2. Provoked Deep Vein Thrombosis

The overwhelming majority of pediatric thromboses are provoked, that is, attributable to an identifiable trigger, most often a central venous catheter and, less commonly, surgery, trauma, infection, immobilization, malignancy, or estrogen-containing contraceptives [44]. The duration of anticoagulation is stratified by the risk of recurrence. Children with a first, low-risk provoked DVT, defined by the absence of prior VTE, a thrombus that is neither severe nor life-threatening, no accompanying pulmonary embolism, a transient provoking factor that has resolved, and a thrombus that has resolved or become non-occlusive within six weeks, may be treated with a six-week course rather than the conventional three months [44,45].

This abbreviated duration is supported by the Kids-DOTT randomized trial, in which 417 patients younger than 21 years with low-risk provoked VTE were assigned to six weeks or three months of anticoagulation; symptomatic recurrence (well below 1 percent in each arm) and clinically relevant bleeding were low and statistically indistinguishable, establishing the non-inferiority of the shorter course [45]. For children who do not meet low-risk criteria, including those with persistent provoking conditions such as malignancy or systemic lupus erythematosus and those with severe or life-threatening thrombosis, at least three months of anticoagulation is recommended, a practice extrapolated largely from adult evidence [44].

#### 2.9.3. Special Situations in Provoked Thrombosis

Catheter-associated thrombosis. Anticoagulation is combined with a decision about the catheter itself. A functioning catheter that is still needed may be left in place while anticoagulation is given and the child is monitored, whereas a non-functioning or unnecessary catheter, or one associated with symptomatic thrombosis, should be removed, ideally after three to five days of anticoagulation; if the catheter remains in place at the end of the treatment course, prophylactic-dose anticoagulation is continued until it is removed [44].

Malignancy. Thrombosis in children with cancer requires special handling because of concurrent central venous catheters, chemotherapy-related shifts in both bleeding and clotting risk, and frequent invasive procedures. Its management is addressed in a dedicated section of this review [44].

Estrogen-containing contraceptives. When VTE arises in an adolescent using combined hormonal contraception, switching to a progestogen-only pill, implant, or levonorgestrel-releasing intrauterine device is generally favored, as these are not associated with increased thrombotic risk and help control the heavy menstrual bleeding that is common during anticoagulation [44].

Systemic lupus erythematosus and antiphospholipid syndrome. Persistent antiphospholipid antibodies generally call for prolonged, often indefinite, anticoagulation, typically with a vitamin K antagonist, because these antibodies may interfere with coagulation-based monitoring assays; chromogenic anti-factor Xa or factor-specific assays should be used when necessary [44].

#### 2.9.4. Unprovoked Venous Thromboembolism

Unprovoked thrombosis, occurring in the absence of any identifiable trigger, is uncommon in children and is treated for a longer period, generally six to twelve months, with indefinite anticoagulation reserved for recurrent unprovoked events [44]. Children with unprovoked VTE warrant evaluation for inherited thrombophilia, since a positive result may influence the duration of therapy [11,44].

#### 2.9.5. Pulmonary Embolism

Pulmonary embolism is treated along the same lines as deep vein thrombosis: an initial parenteral anticoagulant for five to ten days followed by ongoing therapy, with a usual duration of three months for provoked events and six to twelve months for unprovoked events. Thrombolysis or, rarely, thrombectomy is reserved for extensive embolism producing hemodynamic compromise and is undertaken only in consultation with a pediatric hematologist [44].

#### 2.9.6. Limb- or Organ-Threatening Thrombosis

Rarely, a major-vessel thrombosis acutely threatens a limb or vital organ. In these circumstances systemic or, preferably, catheter-directed thrombolysis may be warranted, with surgical or mechanical thrombectomy reserved for thrombolysis failure, although experience with these interventions in children is very limited [44].

### 2.10. Choice of Anticoagulant Agent

Selection of an agent is individualized, taking into account the child’s age, comorbidities, drug interactions, and bleeding risk, together with the practicalities of administration and monitoring and the preferences of the patient and family [44].

#### 2.10.1. Initial Parenteral Therapy

For initial treatment, at least five days of a parenteral anticoagulant is recommended, usually LMWH; UFH is preferred when rapid titratability is required or in severe renal impairment, and fondaparinux is an occasional alternative [44]. The requirement for a parenteral lead-in reflects the design of the pediatric direct oral anticoagulant (DOAC) trials, in which most participants received several days of parenteral therapy before transitioning to an oral agent [44,46,47].

#### 2.10.2. Subsequent Therapy According to Age

Adolescents (12 years and older). A DOAC, apixaban, dabigatran, or rivaroxaban, is generally preferred for continuation therapy, because oral administration and the absence of routine laboratory monitoring are advantageous and efficacy and bleeding risk are comparable to those of LMWH or a vitamin K antagonist [44,46,47].

Children 1 to under 12 years. Either a DOAC or LMWH is reasonable; the choice rests on comorbidities, drug availability and cost, and family and clinician preference, with DOAC use increasing in this age group [44].

Infants under 12 months. LMWH is generally preferred because experience with DOACs is limited and few infants were enrolled in the pivotal trials, although cautious DOAC use is acceptable in selected circumstances; vitamin K antagonists are poorly suited to infants because dietary vitamin K intake varies widely and warfarin is available only in tablet form [44,46,47].

#### 2.10.3. Special Circumstances

Active cancer. Data on DOACs in children with cancer are limited, and many clinicians favor LMWH, although a DOAC may be reasonable for an older adolescent on the basis of subgroup and adult evidence [44,48].

Renal impairment. LMWH and DOACs should be avoided when the estimated glomerular filtration rate falls below 30 mL/min, where warfarin is generally preferred; LMWH may be used with dose reduction and anti-factor Xa monitoring in milder impairment [44].

Heavy menstrual bleeding. Postmenarchal girls face a higher risk of heavy menstrual bleeding with DOACs, greatest with rivaroxaban, which should be addressed as part of shared decision-making and may prompt a change of agent [44].

Antiphospholipid syndrome and mechanical heart valves. Warfarin is the agent of choice; DOACs should not be used in patients with mechanical heart valves [44].

Heparin-induced thrombocytopenia. A non-heparin anticoagulant, such as fondaparinux, argatroban, or bivalirudin, is required, and all heparin exposure, including catheter flushes, must be stopped [44].

### 2.11. Anticoagulant Agents

The anticoagulants most often used to treat VTE in children are low-molecular-weight heparin (LMWH), unfractionated heparin (UFH), the direct oral anticoagulants (DOACs), and the vitamin K antagonists (VKAs). Their principal features are compared in Table 2, and age-based dosing of the principal agents is summarized separately (Table 3). Fondaparinux, bivalirudin, and argatroban play more limited roles [44].

#### 2.11.1. Low-Molecular-Weight Heparin

LMWH is the most widely used parenteral anticoagulant in children, valued for its predictable dose response, subcutaneous administration, and well-established safety [44,49]. Treatment is monitored with the anti-factor Xa assay, targeting 0.5–1.0 units/mL in a sample drawn four to six hours after dosing; neonates and critically ill children typically require higher weight-based doses to reach this target [49,50]. A systematic review of 49 studies encompassing 3101 children confirmed that LMWH is effective, with a low rate of major bleeding of approximately 2–3 percent [49]. If serious bleeding occurs, protamine sulfate neutralizes much of the anti-factor Xa activity, and at least two doses should be withheld before lumbar puncture or neuraxial anesthesia [44].

#### 2.11.2. Unfractionated Heparin

Unfractionated heparin is given as a continuous intravenous infusion at an age-dependent dose, higher in infants because of faster clearance and a larger volume of distribution, and is monitored by anti-factor Xa activity, with a therapeutic target of 0.35–0.7 units/mL [44,51]. The activated partial thromboplastin time correlates poorly with anti-factor Xa levels in young children and is an unreliable sole guide to dosing, so anti-Xa-based titration is preferred [51,52]. Bleeding rates vary widely with illness severity and are highest in critically ill children; other complications include heparin-induced thrombocytopenia and, with prolonged use, reduced bone density, while protamine sulfate provides rapid reversal [44].

#### 2.11.3. Direct Oral Anticoagulants

The direct oral anticoagulants comprise the direct thrombin inhibitor dabigatran and the factor Xa inhibitors rivaroxaban, apixaban, and edoxaban; dabigatran, rivaroxaban, and apixaban are the best studied and are approved for the treatment and secondary prevention of pediatric VTE [44,46,47]. Two large, randomized trials established their role. In EINSTEIN-Jr, 500 children with VTE were assigned to rivaroxaban or standard anticoagulation; recurrent thrombosis was infrequent in both arms (1 versus 3 percent), bleeding rates were similar, and complete thrombus resolution was somewhat more frequent with rivaroxaban [46]. In DIVERSITY, 267 children received dabigatran or standard therapy, with comparable rates of thrombus resolution, recurrence, and major bleeding [47]. Dabigatran has also proved safe for secondary prevention in children [53], and apixaban demonstrated comparable efficacy and tolerability in a pediatric treatment trial [44]. Across these trials, major or clinically relevant bleeding occurred in 1–3 percent and minor bleeding in roughly 20 percent of participants, rates similar to those of comparator anticoagulants, although infants and very young children remained underrepresented [46,47,53]. Routine laboratory monitoring is unnecessary; severe bleeding is managed by stopping the drug and administering an antifibrinolytic agent together with prothrombin complex concentrate, and idarucizumab is available for dabigatran reversal in adults although it has not been formally validated in children [44].

#### 2.11.4. Vitamin K Antagonists

Warfarin is initiated at approximately 0.2 mg/kg (maximum 5 mg), with a lower dose in hepatic impairment or when used for prophylaxis, and is monitored by the international normalized ratio (INR), targeted to 2.0–3.0 for most indications. Children require more frequent INR monitoring and dose adjustment than adults because dietary vitamin K intake varies widely and many concomitant medications alter warfarin metabolism; despite this, major bleeding is uncommon. Reversal is achieved with vitamin K, supplemented by prothrombin complex concentrate or fresh-frozen plasma when bleeding is significant [44].

#### 2.11.5. Other Agents

Fondaparinux, bivalirudin, and argatroban play limited roles in pediatric practice. Fondaparinux is a subcutaneously administered agent occasionally used as an alternative to LMWH for initial therapy, whereas bivalirudin and argatroban are intravenous direct thrombin inhibitors employed chiefly when heparin must be avoided, as in heparin-induced thrombocytopenia [44,54].

#### 2.11.6. Regulatory Status and Formulation-Related Dosing Considerations

Licensing of anticoagulants for children differs by agent and by geographical area, and this has practical consequences for prescribing. Among the direct oral anticoagulants, rivaroxaban and dabigatran were the first agents licensed for the treatment and secondary prevention of pediatric venous thromboembolism, and both carry approvals from the United States Food and Drug Administration and from the European Medicines Agency on the basis of the EINSTEIN-Jr and DIVERSITY trials, with apixaban approved more recently [44,46,47]. The traditional agents, namely low-molecular-weight heparin, unfractionated heparin, and vitamin K antagonists, are used in children largely according to guideline recommendations that extrapolate from adult experience and from pediatric pharmacokinetic studies rather than from broad pediatric labeling, and their approved indications and availability vary from one country to another [44]. Clinicians should therefore confirm which agents and indications are locally licensed before prescribing.

Even when an agent is licensed, choosing and delivering the correct dose is frequently constrained by the commercial formulations that happen to be available. Rivaroxaban can be given as an oral suspension or as tablets, which permits body-weight-adjusted dosing across the pediatric age range, and dabigatran is supplied as age- and weight-adjusted coated pellets and as an oral solution suitable for younger children [46,47]. Warfarin, by contrast, is available only as tablets, so that accurate dosing in small children requires splitting or crushing; no standard pediatric liquid preparation exists, and frequent monitoring of the international normalized ratio remains necessary [44]. Low-molecular-weight heparins are supplied in prefilled syringes and concentrated vials designed for adults, and measuring the very small volumes required for neonates and infants can be difficult and may call for dilution, which introduces a further source of dosing error [44]. Together with the developmental changes in hemostasis discussed above, these formulation-related limitations help explain why anticoagulant dosing in the youngest patients remains one of the most demanding aspects of their care.

### 2.12. Thrombolytic Therapy

Systemic or catheter-directed thrombolysis is restricted to thromboses that imminently threaten a limb or vital organ, recombinant tissue plasminogen activator (alteplase) being the usual agent [44,55]. Catheter-directed delivery is generally preferred because it achieves higher response rates with fewer major bleeding complications than systemic infusion [55]. Thrombolysis is contraindicated in children with right-to-left cardiac shunts because of the risk of systemic and cerebral arterial embolism; bleeding is the principal complication, so predisposing abnormalities should be corrected and fibrinogen monitored before and during treatment, with consultation of a pediatric hematologist or vascular specialist advised [44,55].

### 2.13. Prevention

#### 2.13.1. Primary Prophylaxis in Hospitalized Children

Indications for primary thromboprophylaxis in children without prior VTE are not well established, and the supporting pediatric data are limited; decisions rest on the number and nature of risk factors, whether they are transient or persistent, and the bleeding risk [44,56]. Mechanical measures, graduated compression stockings and, where size permits, intermittent pneumatic compression, together with early mobilization, are encouraged for children with risk factors and are the mainstay when bleeding risk is high, though appropriately sized devices are often unavailable for small children [44]. Pharmacologic prophylaxis, usually with low-molecular-weight heparin, is reserved for children with multiple risk factors and acceptable bleeding risk [44,56]. Recognized inpatient risk factors include a prior VTE or known thrombophilia (each a particularly strong predictor), critical illness, major surgery, trauma, mechanical ventilation, a central venous catheter, systemic infection, prolonged immobility, an anticipated admission of three or more days, postpubertal age, obesity, estrogen-containing contraceptive use, and active inflammatory bowel disease [13,35,57].

Several pediatric risk-prediction models and decision rules have been developed to guide prophylaxis, but none have yet been prospectively validated [13,35,57,58]. A randomized trial of early enoxaparin prophylaxis against catheter-associated thrombosis in critically ill children was stopped early and was inconclusive overall, although a benefit was suggested in older children, underscoring the uncertain risk–benefit balance in this population [59]. Crucially, the routine anticoagulant prophylaxis applied to hospitalized adults should not be extended wholesale to children, in whom the far lower baseline risk makes the risk-benefit ratio less favorable; pharmacologic prophylaxis is generally not warranted when a central venous catheter is the only risk factor [44,59]. The main thromboprophylaxis approaches across the pediatric populations covered in this review are brought together for comparison (Table 4).

Because the indwelling central venous catheter is the single most important and most modifiable contributor to pediatric venous thromboembolism, primary prevention also rests on device- and technique-related measures applied at the time of insertion. Selecting the smallest catheter and the fewest lumens that meet the clinical need, keeping the catheter-to-vein diameter ratio low, positioning the tip at the cavoatrial junction, limiting the number of puncture attempts, and reassessing the ongoing need for the line so that it is removed as early as possible all help to lower the thrombotic burden [5,20]. Real-time ultrasound guidance is preferred over the anatomical landmark technique and over open surgical venesection, because it improves first-pass success and has been associated with lower rates of catheter-related thrombosis in both adult and pediatric series [22,23].

#### 2.13.2. Long-Term Primary Prophylaxis

Long-term antithrombotic prophylaxis may be warranted for children with persistent, substantial thrombotic risk, for example, those dependent on long-term home parenteral nutrition, those undergoing chronic hemodialysis through an arteriovenous fistula or catheter, selected children with congenital nephrotic syndrome, and those with certain cardiac conditions such as single-ventricle (Fontan) physiology or mechanical heart valves [44,67,68]. Warfarin has historically been used and remains the preferred agent for mechanical heart valves, whereas DOACs are increasingly used off-label and antiplatelet therapy with aspirin is an alternative in selected cardiac settings [44,68].

#### 2.13.3. Secondary Prevention

For children who have already experienced a VTE, long-term secondary prophylaxis is suggested when a provoking factor other than a catheter persists or recurs, such as the chronic conditions noted above, inflammatory bowel disease flares, or active systemic lupus erythematosus with persistent antiphospholipid antibodies, and for recurrent unprovoked VTE [44]. In catheter-associated thrombosis, retention of the catheter is itself a risk factor for recurrence, and prophylactic-dose anticoagulation is continued for as long as the catheter remains in place [44,69].

### 2.14. Outcome

#### 2.14.1. Recurrence

Reported rates of recurrent VTE after a pediatric event range widely, from about 1 to 20 percent, depending on the population studied and how recurrence is defined [44,45]. Recurrence is uncommon after provoked thrombosis once the provoking factor resolves, well under 1 percent in the low-risk Kids-DOTT cohort, but is substantially higher after unprovoked events, particularly in children found to have inherited thrombophilia, in whom the risk may be several-fold greater [11,45].

#### 2.14.2. Post-Thrombotic Syndrome

Post-thrombotic syndrome (PTS), chronic venous insufficiency manifesting as limb swelling, pain, skin changes and, in severe cases, ulceration, is the most frequent long-term complication of extremity deep vein thrombosis [44,70]. Its reported incidence varies considerably with the population and the diagnostic instrument used. An ISTH guidance synthesis of pooled data from 1708 children with extremity DVT estimated an overall PTS rate of about 42 percent, with individual-study estimates ranging from 16 to 65 percent [70], whereas a prospective cohort of 294 children assessed with the Manco-Johnson instrument found signs of PTS in 40 percent at one year, including chronic limb pain in 13 percent [71]. Complete venous occlusion, residual thrombus at the end of therapy, and non-catheter-associated DVT are consistent risk factors [70,71]. Several pediatric PTS scoring tools—the modified Villalta scale, the Manco-Johnson instrument, and the CAPTSure score—show good inter-rater reliability, but none have been prospectively validated [70].

#### 2.14.3. After Pulmonary Embolism

Long-term outcomes after pediatric pulmonary embolism are generally favorable. In a cohort of 150 children and adolescents followed after PE, mortality was about 5 percent, recurrence 9 percent, and thrombus non-resolution 29 percent; chronic dyspnea was reported by roughly 7 percent, and although approximately one-third of those tested had abnormal pulmonary-function or exercise testing, most such children had significant underlying chronic disease [72].

#### 2.14.4. Mortality

Overall mortality among children with VTE is historically reported at 8 to 17 percent. Older series that included massive pulmonary embolism described case-fatality as high as 10 to 20 percent, whereas contemporary cohorts report a considerably lower mortality of around 5 percent after pulmonary embolism. These figures are heavily confounded by the serious underlying conditions, malignancy, and cardiac disease, present in most patients. Death attributable directly to thrombosis occurs in only about 2 to 4 percent of cases, most often from pulmonary embolism [44,72].

## 3. Neonatal Thrombosis

### 3.1. Hemostasis in the Newborn

The hemostatic system of the newborn differs substantially from that of older children and adults, a physiologic state termed developmental hemostasis, that strongly influences both bleeding and thrombotic risk in early life [12,73]. At birth, the vitamin K-dependent procoagulant factors (II, VII, IX, and X) and the contact factors (XI, XII, prekallikrein, and high-molecular-weight kininogen) are present at only about 50 to 70 percent of adult levels and rise toward adult values over the first months of life, whereas factors V and VIII, von Willebrand factor, and fibrinogen are at or near adult concentrations [12,73]. The natural anticoagulants antithrombin, heparin cofactor II, and proteins C and S are likewise reduced to roughly half of adult levels, a deficit only partly offset by elevated concentrations of α_2_-macroglobulin, while the overall capacity to generate thrombin is also diminished [12,73]. The fibrinolytic system is similarly rebalanced, with low plasminogen and comparatively high plasminogen activator inhibitor-1 [73]. These differences are even more pronounced in preterm infants, whose lower levels of both procoagulant factors and natural inhibitors leave them especially vulnerable to hemorrhagic and thrombotic complications when challenged by indwelling catheters or critical illness [12,73].

### 3.2. Incidence

Thrombosis is rare in the general newborn population, with population-based estimates of approximately 3 to 5 events per 100,000 live births [7]. Among infants admitted to a neonatal intensive care unit (NICU), the frequency is far higher, on the order of 0.7 to 1.5 percent of admissions, reflecting the concentration of indwelling catheters, critical illness, and prematurity in this setting [74,75]. Contemporary incidence estimates exceed those of registries from the 1990s and early 2000s, a rise attributed both to improved detection and to the greater survival of extremely preterm infants [74]. In a multicenter cohort of nearly 40,000 neonates cared for in 30 Canadian NICUs between 2014 and 2016, 1.5 percent had at least one documented thrombosis; about three-quarters of the events were venous (most often portal vein thrombosis), roughly one-fifth were arterial (most often arterial ischemic stroke), and the remainder were mixed [74].

### 3.3. Risk Factors

Neonatal thrombosis is almost always multifactorial, developing when the immature hemostatic system is exposed to one or more acquired insults. The principal risk factors include the following:

Vascular catheters. Indwelling central venous and arterial catheters are the dominant risk factor for neonatal thrombosis [74,76]. Thrombosis develops in up to roughly 10 percent of neonates with a central venous catheter, most often without symptoms, and a systematic review found a pooled rate of catheter-related arterial thrombosis of about 20 percent among neonates and children with arterial catheters [76,77]. The risk is influenced by catheter dwell time and tip position, with a femoral venous location appearing to carry the greatest risk [75,78].

Other clinical factors. Polycythemia, systemic infection, major surgery, perinatal asphyxia, and underlying disorders such as congenital heart disease, congenital nephrotic syndrome, and certain inborn metabolic diseases all increase thrombotic risk [75].

Inherited thrombophilia. The thrombophilias most clearly linked to thrombosis, deficiencies of antithrombin, protein C, or protein S and the factor V Leiden and prothrombin G20210A variants, may contribute, but their true prevalence among neonates with thrombosis and their causal weight relative to acquired factors remain uncertain [11].

### 3.4. Clinical Features

The clinical presentation of neonatal thrombosis is highly variable and depends on the location and size of the thrombus; many events are clinically silent and are discovered incidentally. As in older children, the great majority of neonatal thromboses are associated with an indwelling vascular catheter, whereas renal vein thrombosis is the most common non-catheter-related form [74].

#### 3.4.1. Catheter-Associated and Central Venous Thrombosis

Most neonatal thromboses arise in association with central venous catheters, which are placed through the umbilical vein, a major vein such as the jugular, or peripherally as peripherally inserted central catheters [74,76]. They are frequently asymptomatic or present only as loss of catheter patency. When symptomatic, they may cause swelling or discoloration of the affected extremity [76]. Thrombosis of the inferior vena cava typically produces swelling of the lower body, whereas superior vena cava thrombosis causes swelling of the arm, neck, and head and may present with chylothorax [79]. A catheter tip lying within the right atrium can give rise to an intracardiac thrombus that manifests as a new murmur, signs of heart failure, or catheter malfunction [76]. Occlusive deep vein thrombosis can lead to post-thrombotic syndrome, although this sequela is less common after neonatal thrombosis than after thrombosis in older children [74].

#### 3.4.2. Portal Vein Thrombosis

Portal vein thrombosis in the neonate is characteristically associated with umbilical venous catheterization [41,80]. Most cases are asymptomatic and resolve spontaneously: in surveillance studies that screened all infants with umbilical venous catheters, reported rates ranged widely, from roughly one-fifth to three-quarters of infants, yet spontaneous resolution within the first year exceeded 95 percent [80]. A minority of infants nonetheless develop long-term sequelae such as hepatic lobar atrophy or portal hypertension; in one cohort of 74 neonates, 60 percent showed complete resolution on serial imaging and only about 4 percent experienced a complication, whereas an earlier series reported higher rates of lobar atrophy [81]. The determinants of these complications remain poorly defined [41,81].

#### 3.4.3. Renal Vein Thrombosis

Renal vein thrombosis (RVT) accounts for roughly 10 percent of neonatal venous thromboses and is the most common form not related to a vascular catheter [40]. It is thought to arise from a combination of reduced renal blood flow, hyperosmolality, hypercoagulability, and increased blood viscosity, and is associated with prematurity, perinatal asphyxia, dehydration, sepsis, polycythemia, cyanotic congenital heart disease, and maternal diabetes, while inherited thrombophilia is more prevalent in affected infants than in the general population [40,82]. The classic triad of a flank mass, hematuria, and thrombocytopenia is present in only a minority of cases, often around one in eight, so a high index of suspicion is required [40]. RVT is unilateral in approximately 70 percent of infants (more often the left kidney), may extend into the inferior vena cava in nearly half, and can be accompanied by adrenal hemorrhage; recognized long-term sequelae include systemic hypertension and chronic kidney disease [40,82]. On Doppler ultrasonography, the appearance of RVT evolves over the first weeks of life, and the sonographic findings can help predict the eventual renal outcome [83].

#### 3.4.4. Arterial Thrombosis

Nearly all arterial thromboses in neonates are associated with indwelling arterial catheters, umbilical, peripheral, or femoral, used for blood-pressure monitoring, blood sampling, or cardiac catheterization [77]. A systematic review estimated the pooled incidence of catheter-related arterial thrombosis at about 20 percent, with reported rates for umbilical arterial catheters of roughly 8 to 20 percent; most are asymptomatic, but presentations range from a pale, cool, poorly perfused extremity to systemic hypertension and, depending on the location of the thrombus, organ ischemia [77,84]. Rare but serious complications of umbilical arterial catheter thrombosis include renal failure, necrotizing enterocolitis, and spinal cord infarction when the thrombus extends into the renal, mesenteric, or spinal arterial branches, and severe peripheral arterial thrombosis can impair subsequent growth of the affected limb [84].

#### 3.4.5. Association with Thrombocytopenia

Neonatal thrombosis is frequently accompanied by thrombocytopenia; the diagnosis of thrombosis should therefore be considered in any neonate with an otherwise unexplained fall in platelet count [75].

#### 3.4.6. Neonatal Purpura Fulminans

Neonatal purpura fulminans is a rare, life-threatening emergency characterized by disseminated intravascular coagulation with widespread venous and arterial thrombosis and hemorrhagic skin necrosis, most often caused by homozygous or compound-heterozygous deficiency of protein C or protein S [85]. It typically presents within the first 24 h of life with ecchymoses and rapidly progressive skin lesions accompanied by laboratory evidence of consumptive coagulopathy and extremely low, often less than 1 percent, protein C or protein S activity [85,86]. Diagnosis rests on measuring protein C and S activity, interpreted against age-specific reference ranges and ideally confirmed by genetic testing, on a sample obtained before treatment; however, empiric fresh-frozen plasma should not be delayed while results are awaited [85].

### 3.5. Diagnosis

Doppler ultrasonography is the imaging modality of choice for confirming venous or arterial thrombosis in the newborn, being noninvasive, free of ionizing radiation, and feasible at the bedside; alternatives such as contrast angiography, magnetic resonance imaging, and computed tomography are rarely required [40,76]. As in older patients, non-compressibility of the vessel, with or without visible intraluminal thrombus, confirms the diagnosis, but interpretation is more difficult in neonates: an indwelling catheter itself reduces vessel compressibility and may obscure thrombus, and the low pulse pressure of preterm and critically ill infants can make assessment of arterial flow challenging [76]. In renal vein thrombosis the sonographic appearance changes over time, from echogenic streaks and a swollen, echogenic kidney in the first days to later loss of corticomedullary differentiation, and color Doppler may demonstrate absent intrarenal and renal venous flow [83].

### 3.6. Additional Testing

The value of thrombophilia testing in a neonate with thrombosis is limited. Because most events are catheter-related and the risk of recurrence during childhood is low, with any later event typically deferred until adolescence or adulthood, testing is reserved for infants with recurrent or unprovoked thrombosis and is best undertaken in consultation with a pediatric hematologist. When testing is performed it is generally deferred until the infant is older, because functional assays are difficult to interpret in the neonatal period and during acute thrombosis, antiphospholipid antibodies are measured in a maternal rather than a neonatal sample, and all level-based results must be compared against postnatal- and gestational-age-specific reference ranges, with abnormal values repeated after several weeks. When anticoagulation is being considered, a baseline evaluation, complete blood count, coagulation screen and fibrinogen, renal and, if a direct oral anticoagulant is planned, hepatic function, together with a cranial ultrasound to exclude intracranial or intraventricular hemorrhage is advisable, particularly in preterm infants [11].

### 3.7. Management-General Approach

Because the prothrombotic insults that precipitate neonatal thrombosis, chiefly indwelling catheters and critical illness, are usually transient, the risk of recurrence is low, and the principal aim of treatment is to prevent thrombus extension and limit end-organ damage rather than to prevent recurrence [44]. Management must be individualized, weighing the often-uncertain benefit of anticoagulation against a meaningful risk of bleeding in a population for which high-quality efficacy and safety data are scarce; many asymptomatic thrombi resolve with catheter removal and supportive care alone [44,87]. The minority of infants with a chronic prothrombotic condition, such as severe congenital protein C or S deficiency or congenital nephrotic syndrome, require longer-term therapy. Involvement of a pediatric hematologist is advised in most cases [44].

### 3.8. Management According to Thrombus Location

The choice between conservative management, supportive care with serial imaging, and anticoagulation depends on the anatomic location of the thrombus and the presence of associated symptoms.

#### 3.8.1. Catheter-Associated Venous Thrombosis

For catheter-associated venous thrombosis outside the renal, portal, and right atrial circulations, asymptomatic clots are generally managed conservatively: the associated central or umbilical venous catheter is removed when feasible, and the thrombus is followed by ultrasonography initially at three to five days and thereafter at lengthening intervals [44,87]. Most such thrombi resolve without anticoagulation, particularly once the catheter is removed, and conservative management spares the infant the bleeding risk of anticoagulation; therapy is reserved for thrombi that extend or become symptomatic [44,87]. Symptomatic catheter-associated thrombosis is treated with therapeutic anticoagulation, usually beginning with low-molecular-weight heparin (LMWH) [44]. The optimal duration is uncertain and is guided by serial imaging: anticoagulation is stopped once the thrombus has resolved and the infant is asymptomatic, generally after six weeks and for no longer than three months, and randomized data in older children indicate that six weeks suffices for most catheter-associated events [45]. A catheter implicated in thrombosis should be removed, typically after three to five days of anticoagulation, and if it must remain in situ, prophylactic-dose LMWH is continued until it is withdrawn [44].

#### 3.8.2. Renal Vein Thrombosis

The optimal management of neonatal renal vein thrombosis (RVT) is uncertain and rests on small retrospective series. The approach is stratified by laterality, kidney function, and whether the thrombus extends into the inferior vena cava (IVC), and any catheter lying within the IVC is removed [44]. For unilateral RVT with preserved kidney function and no IVC extension, anticoagulation is reasonable to limit the long-term risks of hypertension and chronic kidney disease, but initial supportive care with close monitoring, escalating to anticoagulation only if the thrombus progresses, is an acceptable alternative given the relatively low complication risk of unilateral disease [44,82]. For bilateral RVT, impaired kidney function, or extension into the IVC, therapeutic anticoagulation for three months is suggested unless contraindicated, and the addition of thrombolysis may be considered when extensive thrombus threatens kidney function [44]. Reported practice is divided; across pooled neonatal RVT data, roughly 60 percent of infants received anticoagulation, 40 percent were managed conservatively, and a high proportion of affected kidneys show atrophy on follow-up regardless of whether heparin was given, underscoring how limited the evidence base remains [40,82].

#### 3.8.3. Portal Vein Thrombosis

Neonatal portal vein thrombosis is almost always related to an umbilical venous catheter, which should be removed when possible [41]. Because most resolve spontaneously, conservative management with serial imaging is appropriate for thrombosis confined to the left portal vein, whereas anticoagulation, typically with LMWH for six weeks to three months, is reserved for thrombus extending into the main portal vein or the IVC [41,44].

#### 3.8.4. Right Atrial Thrombosis

Right atrial thrombi are usually associated with a central venous catheter and may impair cardiac function or embolize to the lungs; the catheter is removed when possible, and anticoagulation, generally beginning with LMWH, is given for six weeks to three months, with thrombolysis added when cardiac function is compromised [88]. A risk-stratified approach is useful: thrombi with low-risk features, small (under about 2 cm), non-pedunculated, and neither mobile nor serpiginous, may be managed conservatively with close surveillance and anticoagulation begun only if they enlarge, but because the critical clot size is age-dependent, even a small thrombus may warrant treatment in a neonate [88,89].

#### 3.8.5. Arterial Thrombosis

Arterial thrombosis is treated when it significantly impairs perfusion of an extremity or vital organ. Any associated arterial catheter is removed, and anticoagulation is begun with LMWH or unfractionated heparin (UFH). A limb-threatening thrombus warrants urgent surgical consultation and a multidisciplinary approach, and thrombolysis, usually with concurrent low-dose UFH, is reserved for limb-, organ-, or life-threatening events, with surgical thrombectomy as an alternative when thrombolysis is contraindicated [44].

### 3.9. Anticoagulant Agents in the Neonate

The general pharmacology, monitoring, and reversal of the anticoagulant classes are described in Section 2.11; the considerations specific to the newborn are summarized here. For most neonates requiring treatment, LMWH is preferred over the alternatives [44].

#### 3.9.1. Low-Molecular-Weight Heparin

LMWH is the agent of choice for most neonatal thromboses because of its predictable subcutaneous absorption, longer half-life, dose-independent clearance, and reduced need for venous access and laboratory monitoring relative to UFH, advantages that matter greatly in infants with poor venous access [44,49]. Neonates require higher weight-based doses than older children to reach therapeutic anti-factor Xa levels: enoxaparin is typically begun at about 1.5 to 1.7 mg/kg every twelve hours in term infants and 2 mg/kg every twelve hours in preterm infants (with a prophylactic dose near 0.75 mg/kg), and preterm infants both require more drug and take longer to reach target [44,49,90]. Therapy is titrated to an anti-factor Xa level of 0.5 to 1.0 units/mL (0.1 to 0.3 units/mL for prophylaxis) drawn four to six hours after a dose, recognizing that these targets were derived from older patients and are not well validated in neonates [44,90]. Within these limits, LMWH appears effective; pooled neonatal data report treatment success in roughly 97 percent, with major bleeding in only about 2 percent and a low rate of clinically apparent bleeding in neonates specifically, and protamine sulfate provides only partial reversal [49,90]. Dosing must be reduced in renal impairment and the drug avoided altogether in severe renal failure [44].

#### 3.9.2. Unfractionated Heparin

UFH is reserved for situations that demand rapid titratability or reversal, severe renal failure, a high bleeding risk, or concurrent thrombolysis, at the cost of an unpredictable response, frequent monitoring, and a dedicated intravenous line [44,91]. A loading dose is usually omitted in neonates, and a maintenance infusion of about 28 units/kg per hour is titrated against both the anti-factor Xa level (target 0.35 to 0.7 units/mL) and the activated partial thromboplastin time, because the newborn’s accelerated heparin clearance and physiologically low antithrombin concentration make any single test unreliable [44,51,91]. When adequate anticoagulation cannot be achieved despite high doses, a low antithrombin level may be responsible, and antithrombin concentrate can be considered, though supporting data are limited and antithrombin is generally avoided in preterm infants because of safety concerns [92]. Because UFH carries greater risks of bleeding, heparin-induced thrombocytopenia, and osteoporosis than LMWH, many clinicians switch to LMWH when treatment is expected to last beyond about two weeks [44,91].

#### 3.9.3. Direct Oral Anticoagulants and Vitamin K Antagonists

Direct oral anticoagulants have only a limited role in the newborn. They have no place in preterm infants, in whom safety and efficacy data are essentially absent, and although rivaroxaban is approved for term neonates who meet eligibility criteria, LMWH remains preferred for the full course in most cases. A DOAC is a reasonable option chiefly when a stable term infant who has completed initial parenteral therapy needs to transition to an oral agent and meets strict eligibility criteria (gestational-age and weight thresholds, clinical stability, established enteral feeding, and prior parenteral anticoagulation), as detailed in Section 2.11 [44,46]. Warfarin is generally avoided in neonates. The vitamin K-dependent factors it targets are already physiologically low, vitamin K stores are marginal (especially in breastfed infants), and its tablet-only formulation and need for frequent INR monitoring make safe dosing impractical [44].

#### 3.9.4. Other Agents

When heparin must be avoided, most often because of suspected heparin-induced thrombocytopenia, a non-heparin anticoagulant such as argatroban or fondaparinux is used; experience in neonates is limited, but argatroban has been studied in children requiring non-heparin anticoagulation [93].

### 3.10. Thrombolytic Therapy

Thrombolysis is rarely required and is restricted to thrombi that occlude a major vessel and critically threaten an organ or limb, because the bleeding risk is high, the decision is best made by a multidisciplinary team, and recombinant tissue plasminogen activator (tPA) is the agent of choice [44,55]. Several features peculiar to the newborn shape its use: the physiologically low plasminogen concentration of neonates limits plasmin generation, so fresh-frozen plasma is given before and during treatment to supply plasminogen, and tPA is typically infused at a low dose (about 0.1 to 0.2 mg/kg per hour) without a bolus, alongside low-dose UFH, with fibrinogen kept above 100 mg/dL and platelets above 50,000/µL [44,55]. Reported neonatal experience is limited to case series, in which complete or partial clot lysis is common but severe bleeding, including intraventricular hemorrhage, occurs in a substantial minority, a risk compounded by the inherent fragility of critically ill newborns [94,95]. Numerous conditions (recent surgery or hemorrhage, prematurity below 32 weeks, sepsis, active bleeding, and uncorrectable thrombocytopenia or hypofibrinogenemia) contraindicate thrombolysis, and surgical thrombectomy is seldom feasible given the small caliber of neonatal vessels [44,95].

### 3.11. Special Circumstances

#### 3.11.1. Neonatal Purpura Fulminans

Severe congenital protein C or protein S deficiency causes neonatal purpura fulminans, which is treated by replacing the deficient anticoagulant protein; heparin and antiplatelet agents are ineffective [85]. Empiric fresh-frozen plasma (10 to 20 mL/kg every twelve hours) is begun before confirmatory results are available, once protein C deficiency is established, a purified protein C concentrate (for example, an initial 100 units/kg followed by 50 units/kg every six hours, titrated to a trough activity of about 50 IU/dL) is preferred where available, while fresh-frozen plasma is used for protein S deficiency [85]. Because the disorder is lifelong, affected infants require indefinite therapy, long-term anticoagulation (LMWH is generally favored over warfarin or a DOAC) with or without protein C supplementation, which can be given subcutaneously; liver transplantation can correct homozygous protein C deficiency in selected cases [85,96].

#### 3.11.2. Congenital Nephrotic Syndrome

Infants with congenital nephrotic syndrome have a heightened risk of venous thromboembolism owing to urinary loss of anticoagulant proteins. When thrombosis occurs, anticoagulation is continued at therapeutic or prophylactic intensity for as long as the nephrotic state persists, and primary prophylaxis is reasonable in infants with severe disease and additional risk factors. Because antithrombin is depleted, substantially higher LMWH doses (for example, enoxaparin near 3 mg/kg per dose) are often required to reach target anti-factor Xa levels [44,97].

### 3.12. Prevention of Catheter-Associated Thrombosis

A continuous low-dose heparin infusion is standard practice to maintain the patency of umbilical and peripheral arterial catheters and is supported by trial data showing a reduced risk of catheter occlusion. A low-dose heparin infusion is likewise commonly used for central venous catheters, where it reduces occlusion but has not been shown to reduce thrombosis or catheter-related sepsis [44]. An obstructed central catheter can often be cleared by instilling a small dose of tPA confined to the catheter’s dead space, as described for older children in Section 2.9 [44].

As in older children, the risk of catheter-associated thrombosis in neonates is also reduced by careful attention to the insertion itself, namely choosing an appropriately small catheter relative to the vessel, minimizing the number of puncture attempts, using ultrasound guidance where feasible, confirming an optimal tip position, and removing the catheter as soon as it is no longer required [5,23].

### 3.13. Outcome

Reported mortality after neonatal thrombosis is substantial, cited at roughly 10 to 30 percent in older and heterogeneous series, but this largely reflects the underlying critical illness rather than the thrombosis itself, and mortality directly attributable to thrombosis is considerably lower [74]. Long-term sequelae depend on the site: most asymptomatic catheter-associated thrombi resolve without consequence, and although occlusive limb deep vein thrombosis can cause post-thrombotic syndrome, clinically significant post-thrombotic syndrome is less common after neonatal than after later-childhood thrombosis [70]. Portal vein thrombosis occasionally leads to hepatic lobar atrophy or portal hypertension [41], whereas renal vein thrombosis carries the greatest long-term burden, with a high proportion of survivors developing chronic kidney disease and a smaller proportion developing systemic hypertension [40,82].

## 4. Thromboembolism in Children with Cancer

### 4.1. Epidemiology and Risk by Malignancy Type

Children with cancer are at substantially higher risk of thrombosis than the general pediatric population, with reported rates of symptomatic VTE of roughly 4 to 8 percent overall, far above the rate in hospitalized children without cancer, and the incidence has risen over recent decades alongside more sensitive imaging and greater use of central venous catheters [98,99]. The risk varies markedly by tumor type (Table 5): reported rates are highest in solid tumors, particularly sarcomas, and in acute lymphoblastic leukemia (ALL), and lowest in central nervous system tumors, with the higher solid-tumor estimates representing the upper end of the published range [98].

In ALL, recognized risk factors include adolescent age, a T-cell immunophenotype, asparaginase and concomitant corticosteroid therapy, anthracycline exposure, inherited thrombophilia, a non-O blood group, and obesity [100,101,102]. Thrombosis in acute myeloid leukemia is less frequent and reflects, in part, hyperleukocytosis and catheter use [103]. In lymphoma, a mediastinal mass and indwelling central catheters are the principal risk factors [104]. In solid tumors, the risk is amplified by the presence of a central venous catheter, the single most common predisposing factor across all pediatric cancers, and by direct vascular invasion or compression (as in Wilms tumor, hepatoblastoma, or a mediastinal mass), metastatic disease, and age over ten years [105].

### 4.2. Clinical Manifestations and Diagnosis

The clinical manifestations of thrombosis in children with cancer mirror those in children without malignancy and depend on the location, extent, and acuity of the thrombus and the age of the patient [98]. Catheter-related thrombosis, usually in the upper venous system, is by far the most common scenario, but thrombosis may also arise in the lower extremities, lungs, liver, kidneys, or central nervous system, where cerebral sinovenous thrombosis is particularly associated with ALL [98,105]. Diagnosis is often more difficult than in other children because thrombotic symptoms overlap with cancer- and treatment-related complications. A headache from cerebral sinovenous thrombosis, for example, may be ascribed to intrathecal chemotherapy, so a high index of suspicion is essential; the imaging approach (compression ultrasonography, CT pulmonary angiography, or magnetic resonance venography according to the suspected site) is otherwise the same as in children without cancer [98].

### 4.3. Treatment

#### 4.3.1. General Principles and Choice of Agent

Anticoagulating a child with cancer is challenging because the same patient is at heightened risk of both thrombosis and bleeding, the latter from chemotherapy-induced thrombocytopenia and coagulopathy and from frequent invasive procedures, and dedicated pediatric evidence is sparse, so management extrapolates from trials in children without cancer and in adults with cancer [44,106]. In the absence of a contraindication such as active bleeding, symptomatic thrombosis is treated. Low-molecular-weight heparin (LMWH) is the preferred initial agent for at least the first five to ten days, being favored over unfractionated heparin, warfarin, and direct oral anticoagulants (DOACs) in this setting [44,106]. For ongoing therapy, the choice between continuing LMWH and transitioning to a DOAC is individualized, and DOACs are increasingly used as experience accrues [44]. Their pediatric oncology evidence base is still limited but growing: in the cancer subgroup of the EINSTEIN-Jr trial (56 children), no recurrence or major bleeding occurred and most thrombi resolved or improved regardless of treatment arm, although treatment interruptions, usually for thrombocytopenia, were common [48]. Two retrospective series totaling 81 children with cancer-associated VTE treated with a DOAC reported major bleeding in about 2.5 percent, clinically relevant non-major bleeding in 10 percent, and recurrent thrombosis in 11 percent, the higher event rates likely reflecting longer follow-up [107,108].

#### 4.3.2. Dosing and Duration

LMWH is dosed as in the general pediatric population, with the practical caveat that it is withheld before invasive procedures; typically, the last dose is given about 24 h beforehand and resumed 12 to 24 h after a minor procedure, and adjusted for thrombocytopenia [44]. The usual treatment duration is at least three months, although a six-week course suffices for low-risk provoked VTE in children generally; the pivotal trial excluded children with active cancer, so a three-month course remains preferred for most cancer-associated events, with extension when a strong ongoing risk factor such as asparaginase persists [44,45].

#### 4.3.3. Anticoagulation in the Setting of Thrombocytopenia

Because thrombocytopenia is common during cancer treatment and compounds the bleeding risk of anticoagulation, dosing is modulated by the platelet count [106]. During the acute phase (the first two to four weeks, when the thrombus is least stable), platelet transfusions are used to keep the count above about 30,000/µL so that full-dose anticoagulation can continue; thereafter, once the thrombus has stabilized, a graded approach is taken: full-dose anticoagulation for platelets above 30,000/µL, half-dose for counts between 20,000 and 30,000/µL, and withholding the drug below 20,000/µL, with close monitoring for bleeding [106].

#### 4.3.4. Asparaginase-Associated Thrombosis

Asparaginase, a cornerstone of ALL therapy, predisposes to thrombosis, including cerebral sinovenous thrombosis, and a thrombotic event during treatment poses a dilemma, since interrupting asparaginase may compromise the chance of cure. One option is to withhold asparaginase, anticoagulate, and resume the drug once the child has stabilized. An alternative is to continue or re-expose to asparaginase under therapeutic or prophylactic anticoagulation, with or without antithrombin supplementation, an approach shown to be feasible and reasonably safe [109].

#### 4.3.5. Asymptomatic Right Atrial Thrombosis

Right atrial thrombi are increasingly found incidentally on the surveillance echocardiography used to monitor for anthracycline cardiotoxicity. Those with low-risk features, small (under about 2 cm), non-pedunculated, and non-mobile, may be managed conservatively, with removal of the central catheter where possible and ongoing monitoring, whereas anticoagulation is warranted for high-risk thrombi or when the catheter must remain in place, as in neonates, the critical clot size is age-dependent, so a small thrombus may still be high-risk in a young child [88,89].

### 4.4. Impact on Outcome

Cancer-associated thrombosis is not merely a complication but an independent marker of worse prognosis. In a cohort of more than 2000 children with ALL, thromboembolism occurred in about 6 percent and was independently associated with reduced five-year overall survival (80 versus 94 percent, adjusted hazard ratio for death 2.61) [110]. Thrombosis-related mortality itself is lower, around 5 percent in a series of 150 children with cancer-associated thrombosis, and most events are not acutely life-threatening: among 283 pediatric cancer patients with thromboembolism, 92 percent of events were non-life-threatening, 6 percent life-threatening, and 2 percent fatal [98,111]. Beyond direct harm, thrombosis carries indirect costs, including anticoagulation-related bleeding, interruption of cancer therapy, and an approximately two-fold higher risk of recurrent thrombosis than in children without cancer [98].

### 4.5. Prevention

#### 4.5.1. Primary Prophylaxis

For most children with cancer, the best preventive strategy is non-pharmacologic, encouraging mobility, vigilant clinical monitoring, and prompt diagnosis and treatment, because routine pharmacologic thromboprophylaxis has not shown meaningful benefit and adds bleeding risk [60]. Neither malignancy itself nor the presence of a central venous catheter is regarded as an indication for prophylactic anticoagulation; prophylaxis is reserved for selected children with additional strong risk factors, such as a prior thrombosis, known thrombophilia, or multiple combined risk factors (for example, a catheter plus asparaginase plus obesity), an approach consistent with International Society on Thrombosis and Haemostasis guidance [60]. Risk-prediction models have been developed for children with ALL, but none have been prospectively validated [112]. A network meta-analysis of the available trials and observational studies concluded that primary thromboprophylaxis is unlikely to confer a meaningful net benefit in the general pediatric oncology population [113], a conclusion reinforced by subsequent randomized evidence. In the PREVAPIX-ALL trial, 512 children with newly diagnosed ALL or lymphoma were assigned to prophylactic apixaban or to no anticoagulation during induction; apixaban did not significantly reduce symptomatic (1.6 versus 2.3 percent) or asymptomatic thrombosis and caused more clinically relevant non-major bleeding [61]. The earlier THROMBOTECT trial, comparing LMWH, antithrombin, and standard care in 949 children with ALL, found numerically but not statistically lower symptomatic VTE with LMWH or antithrombin, similar event-free survival across arms, and poor acceptability of daily injections [62].

#### 4.5.2. Catheter Selection and Placement

Because catheters drive most cancer-associated thrombosis, device choice and positioning matter. Totally implanted ports are associated with less thrombosis than external tunneled lines, and guidelines favor an internal device where feasible; placement on the right side of the upper venous system with the catheter tip at the cavoatrial junction is preferred, and peripherally inserted central catheters carry a higher thrombotic risk than other central catheters [63,64].

#### 4.5.3. Secondary Prevention

For a child who has had cancer-associated VTE, anticoagulation is extended while a clinically important provoking factor such as asparaginase persists, but it is generally stopped once such factors resolve, even if the catheter remains in place. Children with relapsed disease who experienced thrombosis during prior therapy are offered prophylaxis if they are to receive similarly thrombogenic treatment, reflecting the roughly two-fold higher recurrence risk of cancer-associated thrombosis [60].

## 5. Cerebral Sinovenous Thrombosis

### 5.1. Overview and Epidemiology

Cerebral sinovenous thrombosis (CSVT) is an uncommon but serious cerebrovascular disorder in which thrombosis of the dural venous sinuses or cerebral veins impairs venous drainage of the brain. It is the second most common form of venous thromboembolism in children after extremity deep vein thrombosis, and although rare, it is increasingly recognized [114,115]. The reported incidence is approximately 0.4 to 0.7 per 100,000 children per year in older population-based registries, and appears somewhat higher in contemporary United States inpatient datasets, which estimate roughly 11 new cases per million children per year, equivalent to about 1.1 per 100,000 [114,115]. Newborns are the most frequently affected group, accounting for some 30 to 50 percent of all pediatric cases, with a second, smaller concentration among older children and adolescents [115]. Unlike adults, in whom CSVT predominates in females largely because of pregnancy and hormonal exposures, the sexes are affected at roughly similar rates in children [114].

### 5.2. Pathophysiology

The neurologic consequences of CSVT arise through two interrelated, age-independent mechanisms [115,116]. First, obstruction of venous outflow raises cerebral venous and capillary pressure, reduces perfusion, and disrupts the blood–brain barrier, producing vasogenic and subsequently cytotoxic edema that may progress to venous infarction and, in many cases, hemorrhagic transformation. Because these lesions follow venous rather than arterial drainage, they characteristically do not conform to arterial territories, and they may be partly reversible if the affected vein recanalizes. Second, thrombosis of the dural sinuses impairs reabsorption of cerebrospinal fluid through the arachnoid granulations and raises intracranial pressure, an effect that is especially prominent with superior sagittal sinus involvement [115,116].

### 5.3. Risk Factors and Associated Conditions

Pediatric CSVT is typically multifactorial, and the predominant precipitants are strongly age-dependent [115]. Among newborns, acute systemic illness is the dominant setting, including perinatal complications, dehydration, sepsis, and maternal or peripartum factors [115]. In infants and older children, acute infections of the head and neck, most notably mastoiditis, otitis media, sinusitis, and meningitis, are the leading triggers, a pattern distinct from the hormonal and malignancy-related causes that dominate in adults [115,116]. Dehydration, severe anemia including iron deficiency, and head or neck trauma are additional acute contributors [116]. Chronic systemic disorders also predispose to CSVT, particularly nephrotic syndrome, malignancy (especially acute lymphoblastic leukemia treated with asparaginase), and inflammatory bowel disease [115]. Inherited and acquired thrombophilias, such as factor V Leiden, the prothrombin G20210A variant, deficiencies of antithrombin or protein C or protein S, and elevated lipoprotein(a), are identified in a substantial minority of affected children and add to the multifactorial risk [11,115].

### 5.4. Clinical Features

The clinical presentation of CSVT is often nonspecific and varies markedly with age, which contributes to frequent diagnostic delay [115]. In neonates, seizures and a depressed or altered level of consciousness are the most common manifestations, frequently accompanied by lethargy, poor feeding, and other diffuse, nonfocal signs [115,116]. Infants, older children, and adolescents more often present with headache, nausea and vomiting, and lethargy that reflect raised intracranial pressure, and they may show papilledema, a sixth cranial nerve palsy with diplopia, seizures, focal neurologic deficits such as hemiparesis, or impaired consciousness [115,116]. Because headache or the features of an underlying infection can overshadow the diagnosis, a high index of suspicion is essential, particularly in a child with a predisposing condition [115].

### 5.5. Diagnosis

The diagnosis of CSVT rests on neuroimaging that directly demonstrates venous thrombus or the absence of venous flow. Magnetic resonance imaging combined with magnetic resonance venography is the preferred modality in children because it visualizes both the thrombosed sinus and any parenchymal injury without ionizing radiation [116,117]. Computed tomography venography is an accurate alternative when MRI is not readily available, although it entails radiation exposure, whereas unenhanced CT is relatively insensitive and may reveal only indirect signs such as a hyperdense sinus or the empty delta sign [117]. Characteristic findings include thrombus within a sinus or vein, parenchymal edema, venous infarction that does not respect arterial territories, and cortical or juxtacortical hemorrhage. D-dimer testing lacks sufficient sensitivity to exclude the diagnosis in children and should not be relied upon for that purpose. Once CSVT is confirmed, evaluation for an underlying prothrombotic state is frequently undertaken, as discussed in the section on thrombophilia testing [116,117].

### 5.6. Acute Antithrombotic Management

Anticoagulation is the cornerstone of treatment for childhood CSVT and is recommended for most affected children, including many of those with associated venous infarction or a small amount of intracranial hemorrhage, because the benefit of preventing thrombus propagation generally outweighs the bleeding risk [44,116]. In a prospective pediatric safety and outcome study, anticoagulation was well tolerated, and a lack of anticoagulation strongly predicted thrombus propagation, providing much of the rationale for current recommendations [118]. Initial treatment is usually given as low-molecular-weight heparin or unfractionated heparin [44,116]. The principal exception is the neonate, in whom the decision to anticoagulate is more individualized because of competing bleeding risks, although anticoagulation is used increasingly and is reasonable in the absence of significant hemorrhage [116,118].

### 5.7. Choice of Agent and Duration of Treatment

After initial heparinization, ongoing anticoagulation may be continued with low-molecular-weight heparin or transitioned to a vitamin K antagonist, and increasingly to a direct oral anticoagulant in older children [44,119]. The EINSTEIN-Jr cerebral venous thrombosis study, a predefined subgroup of a randomized pediatric trial, found that rivaroxaban and standard anticoagulants produced similarly low rates of recurrent thrombosis and clinically relevant bleeding in children with CSVT, supporting rivaroxaban as an option in suitable patients [119]. The usual duration of treatment is approximately three months, with reassessment by repeat venous imaging, and a longer course of six months or more is considered when a persistent prothrombotic risk factor or incomplete recanalization is present [44,116].

### 5.8. Management of Underlying Conditions and Acute Complications

Effective treatment requires attention to the precipitating illness as well as to the thrombosis. Acute head and neck infections such as mastoiditis and sinusitis must be treated with appropriate antibiotics and, when indicated, surgical drainage, dehydration, and anemia should be corrected [115,116]. Raised intracranial pressure, seizures, and, less commonly, hydrocephalus are managed supportively, with antiepileptic drugs for clinical or electrographic seizures and with monitoring of vision when intracranial hypertension threatens the optic nerves [116].

### 5.9. Endovascular and Surgical Therapy

Endovascular thrombolysis or mechanical thrombectomy and decompressive craniectomy are reserved for the minority of children who deteriorate despite adequate anticoagulation or who have impending herniation, and the supporting evidence is limited to small case series [116].

### 5.10. Monitoring

Children with CSVT are followed with repeat venous neuroimaging to document recanalization and to detect thrombus propagation, which informs decisions about the duration of anticoagulation [116,117]. Persistent failure of recanalization is associated with a less favorable neurologic outcome [120].

### 5.11. Prognosis

The prognosis of pediatric CSVT is more favorable than that of arterial ischemic stroke but remains serious. Reported mortality is on the order of 5 to 10 percent, and death is usually related to the underlying condition or to extensive venous infarction rather than to the thrombosis alone [114,115]. Recanalization is common, occurring in the great majority of neonates within a few months, and recurrent CSVT is less frequent than recurrence of arterial stroke, with the highest recurrence risk among non-neonatal children, those with a persistent thrombophilia, and those in whom the vessel does not recanalize [120,121]. Long-term neurologic sequelae nonetheless affect a substantial proportion of survivors, including motor and cognitive deficits and epilepsy, and the presence of venous infarction or seizures at presentation predicts a poorer outcome [115,120]. In neonates specifically, outcome studies report frequent neurodevelopmental impairment, underscoring the need for structured neurologic and developmental follow-up [121].

## 6. Venous Thromboembolism in the Severely Injured Pediatric Trauma Patient

### 6.1. Epidemiology and Risk Factors

Venous thromboembolism is far less common after trauma in children than in adults. Reported rates in injured children, generally those under 14 years of age, range from about 0.3 to 1.2 percent, so the balance of benefit and bleeding risk for pharmacologic prophylaxis is less favorable than in adults, and adult trauma protocols cannot simply be transferred to children [27,28,29]. Thrombotic risk rises steeply with age, approaching adult rates in postpubertal adolescents, and with increasing injury severity [28,65]. The major risk factors identified in pediatric trauma cohorts include older, postpubertal age, high injury severity, the presence of a central venous catheter, and operative intervention, with additional contributions from critical illness, blood transfusion, mechanical ventilation, prolonged immobility, spinal cord injury, lower-extremity or pelvic fracture, severe obesity, and a prior history of VTE [27,28,29,65].

### 6.2. Clinical Suspicion and Surveillance

The diagnosis of VTE in an injured child can be obscured because the signs of thrombosis overlap with those of the injuries themselves, so a high index of suspicion and prompt imaging are warranted whenever VTE is suspected. Routine surveillance ultrasonography to detect occult thrombosis is not generally recommended for pediatric trauma patients, because there is no clear evidence that screening asymptomatic children improves outcomes [65].

### 6.3. Thromboprophylaxis

Early mobilization and mechanical methods such as graduated compression and, where size permits, intermittent pneumatic compression are encouraged for injured children with risk factors and are the mainstay of prophylaxis when bleeding risk precludes anticoagulants. The decision to add pharmacologic prophylaxis is guided primarily by pubertal status [65,66].

Prepubertal children. Routine pharmacologic thromboprophylaxis is not recommended for prepubertal trauma patients, in whom VTE is rare. It is reserved for the child with several additional high-risk features, such as high injury severity, critical illness, major surgery, transfusion, mechanical ventilation, a central venous catheter, severe obesity, prolonged immobility, or a prior VTE [65,66].

Postpubertal adolescents. Because the thrombotic risk in postpubertal adolescents with major trauma approaches that of adults, this group is generally managed according to adult trauma prophylaxis recommendations [28,65].

This age-based approach is consistent with the joint practice-management guideline of the Eastern Association for the Surgery of Trauma and the Pediatric Trauma Society and with a multidisciplinary consensus statement [65,66].

### 6.4. Risk Prediction Models

Several risk-prediction tools have been developed to identify injured children who might benefit from prophylaxis, although none have yet been prospectively validated [122,123]. These models variably combine age, Glasgow Coma Scale score, injury severity score, sex, major surgery, blood transfusion, critical illness, the presence of a central venous catheter, immobilization, and pelvic or lower-extremity fracture, and they discriminate risk well, with actual VTE rates of roughly 5 to 10 percent among children in the highest predicted-risk strata [65,122,123].

## 7. Special Populations and Emerging Considerations

### 7.1. Infection-Associated Thrombosis: COVID-19 and Multisystem Inflammatory Syndrome in Children

The SARS-CoV-2 pandemic drew renewed attention to infection-associated thrombosis in children. Hospitalized children with acute coronavirus disease 2019 (COVID-19) or the multisystem inflammatory syndrome in children (MIS-C) have an increased risk of venous thromboembolism relative to other hospitalized children, particularly adolescents and those with additional risk factors such as a central venous catheter, obesity, or critical illness [25]. Reflecting the prothrombotic, hyperinflammatory state of these conditions, consensus-based recommendations suggest pharmacologic thromboprophylaxis, usually with low-molecular-weight heparin, for hospitalized children who have markedly elevated D-dimer levels or one or more additional risk factors for hospital-associated thrombosis, in the absence of contraindications [124].

### 7.2. Thrombosis During Extracorporeal Support and Ventricular Assist Devices

Children supported with extracorporeal membrane oxygenation or a ventricular assist device face among the highest thrombotic and hemorrhagic risks in pediatrics, because blood is exposed to large artificial surfaces and normal flow is profoundly altered. Systemic anticoagulation, most often with unfractionated heparin and increasingly with direct thrombin inhibitors such as bivalirudin, is required to keep the circuit and device patent, yet bleeding and device thrombosis remain frequent, and the optimal agents, targets, and monitoring are not well defined. Management is further complicated by developmental hemostasis, difficult vascular access, and a lack of pediatric-specific drug formulations, and it is best delivered by experienced multidisciplinary teams [125].

### 7.3. Antithrombotic Therapy in Congenital Heart Disease and the Fontan Circulation

Congenital heart disease, especially single-ventricle physiology palliated by the Fontan operation, predisposes to both venous and arterial thrombosis through low-flow states, prosthetic material, and chronically elevated venous pressure. Long-term antithrombotic prophylaxis is therefore standard after the Fontan procedure, although whether aspirin or an anticoagulant is preferable remains debated, and warfarin is required for mechanical heart valves [44,126]. Direct oral anticoagulants are increasingly studied in this setting, and a randomized trial supported rivaroxaban as an alternative to standard therapy for post-Fontan thromboprophylaxis [68]. Decisions are individualized to the specific lesion, the presence of prosthetic material, and any prior thrombotic events [126].

### 7.4. Antiphospholipid Syndrome

Antiphospholipid syndrome, defined by thrombosis together with persistently positive antiphospholipid antibodies, is an uncommon but important cause of both venous and arterial thrombosis in children, and it may occur in isolation or in association with systemic lupus erythematosus. Because the antibodies confer an ongoing thrombotic tendency, anticoagulation is typically prolonged and often indefinite, usually with a vitamin K antagonist, and direct oral anticoagulants are generally avoided in high-risk, triple-antibody-positive disease [44,127]. European evidence-based recommendations emphasize confirming persistent antibody positivity and tailoring the intensity and duration of anticoagulation to the thrombotic phenotype [127].

### 7.5. Management of the Post-Thrombotic Syndrome

Beyond its recognition and prevention, established post-thrombotic syndrome requires ongoing management, because affected children may carry its burden for decades. Graduated compression garments, the mainstay of adult care, reduce symptom severity in children as well, although adherence is limited because some children find them uncomfortable or difficult to apply [128]. A structured approach that combines compression, physical activity, skin care, and the use of validated severity scores to guide therapy, delivered through multidisciplinary follow-up, is recommended, while high-quality trials of preventive and therapeutic interventions remain an unmet need [70,128].

### 7.6. Sickle Cell Disease and Chronic Inflammatory and Renal Disease

Several chronic pediatric diseases carry a persistently elevated thrombotic risk that shapes both prophylaxis and treatment. Children and, especially, adolescents with sickle cell disease have a hypercoagulable state, and in a large multicenter cohort roughly 1.7 percent developed venous thromboembolism, with central venous catheters, chronic kidney disease, prior stroke, female sex, and older age as the principal risk factors and an independent association with mortality. Because the strongest modifiable factor is the catheter, judicious use of central venous access and heightened vigilance during hospitalization for painful crises are central to prevention [129]. In nephrotic syndrome, urinary loss of the natural anticoagulants produces an acquired hypercoagulable state, and thromboprophylaxis is considered during severe relapses or when additional risk factors are present [30,44]. Inflammatory bowel disease similarly raises thrombotic risk, particularly during active disease, and both consensus guidance and pediatric cohort data support considering pharmacologic thromboprophylaxis in hospitalized children experiencing a moderate to severe flare [31,32].

### 7.7. Thrombosis After Hematopoietic and Solid-Organ Transplantation

Transplantation imposes a distinctive, procoagulant, and proinflammatory environment. In a multicenter cohort of children undergoing hematopoietic cell transplantation, venous thromboembolism occurred in about 7 percent, was more frequent in adolescents and in recipients of allogeneic grafts, and was associated with graft-versus-host disease, infection, prolonged hospitalization, and increased one-year mortality. Central venous catheters, endothelial injury from conditioning regimens, and the underlying malignancy all contribute, and concurrent thrombocytopenia complicates anticoagulation, so management must be individualized and multidisciplinary [130]. Solid-organ transplantation, particularly of the liver and kidney, likewise predisposes to vascular thrombosis, including hepatic artery, portal vein, and renal vein thrombosis, which are considered elsewhere in this review [30].

### 7.8. Long-Term Cardiopulmonary Sequelae After Pulmonary Embolism

Although chronic thromboembolic pulmonary hypertension is a recognized and feared long-term complication of pulmonary embolism in adults, it appears to be rare in children. In a contemporary cohort of children and adolescents followed after pulmonary embolism, long-term cardiopulmonary outcomes were generally favorable, with chronic dyspnea in only a small minority and abnormal pulmonary-function or exercise testing largely confined to children with significant underlying disease. Nonetheless, because the data are limited and follow-up is often short, structured cardiopulmonary surveillance after significant pulmonary embolism is prudent, with referral for further evaluation when exertional symptoms or objective abnormalities persist [72].

## 8. Conclusions

Venous thromboembolism in the young is no longer a clinical rarity but a recognized and growing complication of advanced neonatal, pediatric, and adolescent care. Across every setting reviewed here, several unifying themes emerge. Thrombosis in children is overwhelmingly multifactorial, and the indwelling central venous catheter is the single most important and most modifiable contributor. The biology of the developing hemostatic system explains both the relative protection of healthy children and the particular vulnerability of the critically ill neonate, and it underlies age-specific differences in presentation, drug dosing, and outcome. Low-molecular-weight heparin remains the foundation of acute treatment, but direct oral anticoagulants have rapidly acquired a pediatric evidence base and now provide an oral option for many patients beyond infancy. Randomized data have begun to refine practice, supporting a shorter course of anticoagulation for low-risk provoked events while cautioning against routine pharmacologic prophylaxis in hospitalized children, including those with cancer or trauma, in whom the balance of risk and benefit differs fundamentally from that in adults. Special populations, namely children with malignancy, neonates with renal or portal vein thrombosis, patients with cerebral sinovenous thrombosis, and the severely injured, each demand individualized, multidisciplinary management. Important gaps nonetheless persist. Much of pediatric practice still rests on extrapolation from adult studies, prospectively validated risk-prediction tools are lacking, and the optimal intensity and duration of treatment and prophylaxis remain uncertain in several settings. The maturation of pediatric randomized trials and international registries promises to replace expert opinion with evidence over the coming decade. Until then, the recognition that childhood thrombosis is often preventable, and that its consequences may be lifelong, should prompt vigilance, structured risk assessment, and the early involvement of clinicians experienced in pediatric thrombosis. As a growing number of survivors of childhood thrombosis and its underlying conditions reach adulthood, structured transition to adult care has become an increasingly important priority.

## Figures and Tables

**Table 1:** Major risk factors for venous thromboembolism in children, organized by the components of Virchow’s triad.

Component of Virchow’s Triad	Representative Risk Factors in Children
Endothelial injury	Central venous catheters, major trauma and surgery (especially cardiac and orthopedic), severe systemic infection and sepsis, vascular anomalies such as May–Thurner syndrome, thoracic outlet (Paget–Schroetter) syndrome, and inferior vena cava atresia
Venous stasis	Prolonged immobility, critical illness and prolonged mechanical ventilation, low-flow states in congenital heart disease (e.g., single-ventricle or Fontan physiology), obesity, extrinsic venous compression
Hypercoagulability	Inherited thrombophilia (factor V Leiden, prothrombin G20210A, and deficiencies of antithrombin, protein C, or protein S), malignancy and asparaginase therapy, estrogen-containing contraceptives, nephrotic syndrome and other protein-losing states, inflammatory and autoimmune disease (inflammatory bowel disease, systemic lupus erythematosus, antiphospholipid syndrome), and acute infection, including COVID-19

**Table 2:** Principal anticoagulant agents used in the treatment of pediatric venous thromboembolism.

Agent Class	Route	Monitoring	Reversal	Key Pediatric Considerations
LMWH	Subcutaneous	Anti-factor Xa (target 0.5–1.0 U/mL for treatment)	Protamine sulfate (partial)	Most widely used, predictable response, higher weight-based doses required in neonates and critically ill children
UFH	Intravenous infusion	Anti-factor Xa (target 0.35–0.7 U/mL), aPTT unreliable in young children	Protamine sulfate	Preferred when rapid titration or reversal is needed, or in severe renal impairment
DOAC (dabigatran, rivaroxaban, apixaban)	Oral	Not routinely required	Idarucizumab for dabigatran (adult data), PCC and antifibrinolytics	Approved for treatment and secondary prevention, limited data in infants, risk of heavy menstrual bleeding
VKA (warfarin)	Oral	INR (target 2.0–3.0)	Vitamin K ± PCC or FFP	Frequent monitoring, diet and drug interactions, preferred for mechanical valves and antiphospholipid syndrome

LMWH, low-molecular-weight heparin; UFH, unfractionated heparin; DOAC, direct oral anticoagulant; VKA, vitamin K antagonist; aPTT, activated partial thromboplastin time; INR, international normalized ratio; PCC, prothrombin complex concentrate; FFP, fresh-frozen plasma.

**Table 3:** Age-based dosing of anticoagulants commonly used in the treatment and prevention of venous thromboembolism in neonates, children, and adolescents. Doses are provided as general guidance, and product labeling and institutional protocols should be consulted, particularly for the weight- and age-banded direct oral anticoagulants.

Agent (Class, Route)	Therapeutic Dosing	Prophylactic Dosing	Target and Monitoring	Age-Specific Notes
Enoxaparin (LMWH, subcutaneous)	Preterm neonates about 2 mg/kg every 12 h, term neonates 1.5 to 1.7 mg/kg every 12 h, young infants under about 3 months often 1.5 mg/kg every 12 h, older infants and children approximately 1 mg/kg every 12 h	About 0.5 mg/kg every 12 h, approximately 0.75 mg/kg every 12 h in neonates	Anti-Xa 0.5 to 1.0 IU/mL for treatment or 0.1 to 0.3 IU/mL for prophylaxis, measured 4 to 6 h after a dose	Preferred agent at all ages including neonates, dose reduced in renal impairment and avoided in severe renal failure
Unfractionated heparin (intravenous)	Loading 75 units/kg, often omitted in neonates, then 28 units/kg per h in infants and 20 units/kg per h in children	Not generally used for pharmacologic prophylaxis	Anti-Xa 0.35 to 0.7 IU/mL, with activated partial thromboplastin time 1.5 to 2 times control as an adjunct in neonates	Preferred when rapid reversibility is needed or in severe renal failure; aPTT unreliable in young children
Dabigatran (DOAC, direct thrombin inhibitor, oral)	Age- and weight-based dosing per product labeling, after at least 5 days of parenteral therapy	Continued at the treatment dose when used for secondary prevention	Routine laboratory monitoring not required	Approved for children beyond infancy following initial parenteral therapy
Rivaroxaban (DOAC, factor Xa inhibitor, oral)	Weight-based dosing per product labeling, after at least 5 days of parenteral therapy	Approximately one-half of the treatment dose	Routine laboratory monitoring not required	Approved including term neonates who meet eligibility criteria such as weight of at least 2.6 kg and established enteral feeding
Apixaban (DOAC, factor Xa inhibitor, oral)	Weight-based dosing per product labeling, after initial parenteral therapy	Approximately one-half of the treatment dose	Routine laboratory monitoring not required	Used mainly in older children and adolescents
Warfarin (vitamin K antagonist, oral)	Initial 0.2 mg/kg once daily, maximum 5 mg	Lower initial dose, about 0.1 mg/kg	International normalized ratio 2.0 to 3.0 for most indications, 2.5 to 3.5 for mechanical heart valves	Contraindicated in neonates, requires frequent INR monitoring, sensitive to dietary and drug interactions

LMWH, low-molecular-weight heparin; DOAC, direct oral anticoagulant; anti-Xa, anti-factor Xa activity; IU, international units.

**Table 4:** Summary of thromboprophylaxis approaches across the pediatric populations.

Population or Setting	Non-Pharmacologic Measures	Pharmacologic Prophylaxis	Key Points
Hospitalized children (general)	Early mobilization, graduated compression stockings, and intermittent pneumatic compression where size permits [44]	Low-molecular-weight heparin reserved for children with multiple risk factors and acceptable bleeding risk [44,56]	Adult protocols should not be applied wholesale, and a catheter alone is generally not an indication [44,59]
Central venous catheter (device and technique)	Smallest adequate catheter and fewest lumens, low catheter-to-vein ratio, ultrasound-guided placement, tip at the cavoatrial junction, and early removal [5,20,21,22,23]	Not routinely indicated when a catheter is the sole risk factor [44,59]	The catheter is the single most modifiable contributor to pediatric venous thromboembolism [5,20]
Neonates with a central catheter	Careful catheter selection and placement, and early removal once no longer needed [5,23]	Low-dose heparin infusion maintains catheter patency but is not proven to reduce thrombosis [44]	Prophylaxis individualized to the critically ill neonate [44]
Children with cancer	Encourage mobility, vigilant monitoring, and prompt diagnosis [60]	Routine prophylaxis not recommended, reserved for additional strong risk factors [60]	Randomized trials did not show a meaningful net benefit [61,62], and totally implanted ports are preferred over external lines [63,64]
Pediatric trauma	Early mobilization and mechanical methods when bleeding risk precludes anticoagulants [65,66]	Guided by pubertal status, not routine in prepubertal children, and postpubertal adolescents managed as adults [28,65,66]	Age-based approach consistent with Eastern Association for the Surgery of Trauma and Pediatric Trauma Society guidance [65,66]
Long-term high-risk conditions	Measures specific to the underlying condition [44]	Long-term prophylaxis, with warfarin for mechanical valves, direct oral anticoagulants increasingly used, and aspirin in selected cardiac settings [44,67,68]	Applies to home parenteral nutrition, chronic hemodialysis, congenital nephrotic syndrome, and Fontan physiology [44,67,68]
Secondary prevention after VTE	Address modifiable provoking factors [44]	Prophylactic-dose anticoagulation while a provoking factor or catheter persists [44,69]	A retained catheter is itself a reason to continue prophylaxis [44,69]

**Table 5:** Reported rates of venous thromboembolism by pediatric malignancy type.

Malignancy	Reported VTE Rate	Notable Associations
Acute lymphoblastic leukemia	3–15%	Asparaginase and corticosteroid therapy, T-cell phenotype, central venous catheter, adolescent age
Acute myeloid leukemia	4–6%	Hyperleukocytosis, central venous catheter
Lymphoma	5–12%	Mediastinal mass, central venous catheter or PICC
Solid tumors	12–19%	Ewing sarcoma, vascular invasion or compression, metastatic disease, age > 10 years
Central nervous system tumors	0.5–3%	Lowest risk among childhood cancers

VTE, venous thromboembolism; PICC, peripherally inserted central catheter.

## Data Availability

No new data were created or analyzed in this study. Data sharing is not applicable to this article.

## Abbreviations

The following abbreviations are used in this manuscript:

ALL	acute lymphoblastic leukemia
anti-Xa	anti-factor Xa activity
aPTT	activated partial thromboplastin time
ASH	American Society of Hematology
COVID-19	coronavirus disease 2019
CSVT	cerebral sinovenous thrombosis
CT	computed tomography
CTPA	computed tomography pulmonary angiography
CVC	central venous catheter
DOAC	direct oral anticoagulant
DVT	deep vein thrombosis
FFP	fresh-frozen plasma
INR	international normalized ratio
ISTH	International Society on Thrombosis and Haemostasis
IU	international unit
IVC	inferior vena cava
LMWH	low-molecular-weight heparin
MIS-C	multisystem inflammatory syndrome in children
MRI	magnetic resonance imaging
MRV	magnetic resonance venography
NICU	neonatal intensive care unit
PCC	prothrombin complex concentrate
PE	pulmonary embolism
PICC	peripherally inserted central catheter
PTS	post-thrombotic syndrome
RVT	renal vein thrombosis
SARS-CoV-2	severe acute respiratory syndrome coronavirus 2
tPA	tissue plasminogen activator
UFH	unfractionated heparin
VKA	vitamin K antagonist
VTE	venous thromboembolism

## References

[1] Mahajerin A., Croteau S.E. (2017). Epidemiology and Risk Assessment of Pediatric Venous Thromboembolism. Front. Pediatr..

[2] Jaffray J., Young G. (2018). Deep Vein Thrombosis in Pediatric Patients. Pediatr. Blood Cancer.

[3] Takemoto C.M., Sohi S., Desai K., Bharaj R., Khanna A., McFarland S., Klaus S., Irshad A., Goldenberg N.A., Strouse J.J. (2014). Hospital-Associated Venous Thromboembolism in Children: Incidence and Clinical Characteristics. J. Pediatr..

[4] O’Brien S.H., Stanek J.R., Witmer C.M., Raffini L. (2022). The Continued Rise of Venous Thromboembolism Across US Children’s Hospitals. Pediatrics.

[5] Jaffray J., Bauman M., Massicotte P. (2017). The Impact of Central Venous Catheters on Pediatric Venous Thromboembolism. Front. Pediatr..

[6] Shoag J., Davis J.A., Corrales-Medina F.F. (2021). Venous Thromboembolism in Pediatrics. Pediatr. Rev..

[7] Tuckuviene R., Christensen A.L., Helgestad J., Johnsen S.P., Kristensen S.R. (2011). Pediatric Venous and Arterial Noncerebral Thromboembolism in Denmark: A Nationwide Population-Based Study. J. Pediatr..

[8] Setty B.A., O’Brien S.H., Kerlin B.A. (2012). Pediatric Venous Thromboembolism in the United States: A Tertiary Care Complication of Chronic Diseases. Pediatr. Blood Cancer.

[9] Carpenter S.L., Richardson T., Hall M. (2018). Increasing Rate of Pulmonary Embolism Diagnosed in Hospitalized Children in the United States from 2001 to 2014. Blood Adv..

[10] Giordano P., Grassi M., Saracco P., Molinari A.C., Gentilomo C., Suppiej A., Indolfi G., Lasagni D., Luciani M., Piersigilli F. (2018). Paediatric Venous Thromboembolism: A Report from the Italian Registry of Thrombosis in Children (RITI). Blood Transfus..

[11] van Ommen C.H., Nowak-Göttl U. (2017). Inherited Thrombophilia in Pediatric Venous Thromboembolic Disease: Why and Who to Test. Front. Pediatr..

[12] Attard C., van der Straaten T., Karlaftis V., Monagle P., Ignjatovic V. (2013). Developmental Hemostasis: Age-Specific Differences in the Levels of Hemostatic Proteins. J. Thromb. Haemost..

[13] Mahajerin A., Branchford B.R., Amankwah E.K., Raffini L., Chalmers E., van Ommen C.H., Goldenberg N.A. (2015). Hospital-Associated Venous Thromboembolism in Pediatrics: A Systematic Review and Meta-Analysis of Risk Factors and Risk-Assessment Models. Haematologica.

[14] Menéndez J.J., Verdú C., Calderón B., Gómez-Zamora A., Schüffelmann C., de la Cruz J., de la Oliva P. (2016). Incidence and Risk Factors of Superficial and Deep Vein Thrombosis Associated with Peripherally Inserted Central Catheters in Children. J. Thromb. Haemost..

[15] Jaffray J., Witmer C., O’Brien S.H., Diaz R., Ji L., Krava E., Young G. (2020). Peripherally Inserted Central Catheters Lead to a High Risk of Venous Thromboembolism in Children. Blood.

[16] Chopra V., Anand S., Hickner A., Buist M., Rogers M.A., Saint S., Flanders S.A. (2013). Risk of Venous Thromboembolism Associated with Peripherally Inserted Central Catheters: A Systematic Review and Meta-Analysis. Lancet.

[17] Patel N., Petersen T.L., Simpson P.M., Feng M., Hanson S.J. (2020). Rates of Venous Thromboembolism and Central Line-Associated Bloodstream Infections Among Types of Central Venous Access Devices in Critically Ill Children. Crit. Care Med..

[18] Kanin M., Young G. (2013). Incidence of Thrombosis in Children with Tunneled Central Venous Access Devices versus Peripherally Inserted Central Catheters (PICCs). Thromb. Res..

[19] Neshat-Vahid S., Pierce R., Hersey D., Raffini L.J., Faustino E.V.S. (2016). Association of Thrombophilia and Catheter-Associated Thrombosis in Children: A Systematic Review and Meta-Analysis. J. Thromb. Haemost..

[20] Fu M., Yuan Q., Yang Q., Yu Y., Song W., Qin X., Luo Y., Xiong X., Yu G. (2024). Risk factors and incidence of central venous access device-related thrombosis in hospitalized children: A systematic review and meta-analysis. Pediatr. Res..

[21] Kahveci F., Çelik N.A., Uçmak H., Gurbanov A., Coşkun M.K., Özen H., Yılmaz Ş., Havan M., Fitöz S., Ertem M. (2025). Impact of catheter-to-vein diameter ratio on thrombosis in pediatric central venous catheterization. Front. Pediatr..

[22] Stokowski G., Steele D., Wilson D. (2009). The use of ultrasound to improve practice and reduce complication rates in peripherally inserted central catheter insertions: Final report of investigation. J. Infus. Nurs..

[23] Kouna N., Noutsos G., Koufopoulou C., Panagopoulos D., Kattamis A. (2023). Open versus transcutaneous (ultrasound-guided and based on anatomic landmarks) tunneled venous access to the right internal jugular vein in children: A prospective single-center study. Diseases.

[24] Chalmers E., Ganesen V., Liesner R., Maroo S., Nokes T., Saunders D., Williams M. (2011). Guideline on the Investigation, Management and Prevention of Venous Thrombosis in Children. Br. J. Haematol..

[25] Whitworth H., Sartain S.E., Kumar R., Armstrong K., Ballester L., Betensky M., Cohen C.T., Diaz R., Diorio C., Goldenberg N.A. (2021). Rate of Thrombosis in Children and Adolescents Hospitalized with COVID-19 or MIS-C. Blood.

[26] Cheng R., Wang Q., Jiang L., Liu L.-M. (2024). Pulmonary Thromboembolism Due to Mycoplasma pneumoniae in Children: A Case Report and Literature Review. BMC Pediatr..

[27] Thompson A.J., McSwain S.D., Webb S.A., Stroud M.A., Streck C.J. (2013). Venous Thromboembolism Prophylaxis in the Pediatric Trauma Population. J. Pediatr. Surg..

[28] Van Arendonk K.J., Schneider E.B., Haider A.H., Colombani P.M., Stewart F.D., Haut E.R. (2013). Venous Thromboembolism After Trauma: When Do Children Become Adults?. JAMA Surg..

[29] Allen C.J., Murray C.R., Meizoso J.P., Ray J.J., Neville H.L., Schulman C.I., Namias N., Sola J.E., Proctor K.G. (2016). Risk Factors for Venous Thromboembolism After Pediatric Trauma. J. Pediatr. Surg..

[30] Kumar R., Kerlin B.A. (2017). Thrombosis of the Abdominal Veins in Childhood. Front. Pediatr..

[31] Nguyen G.C., Bernstein C.N., Bitton A., Chan A.K., Griffiths A.M., Leontiadis G.I., Geerts W., Bressler B., Butzner J.D., Carrier M. (2014). Consensus Statements on the Risk, Prevention, and Treatment of Venous Thromboembolism in Inflammatory Bowel Disease: Canadian Association of Gastroenterology. Gastroenterology.

[32] Kuenzig M.E., Bitton A., Carroll M.W., Kaplan G.G., Otley A.R., Singh H., Nguyen G.C., Griffiths A.M., Stukel T.A., Targownik L.E. (2021). Inflammatory Bowel Disease Increases the Risk of Venous Thromboembolism in Children: A Population-Based Matched Cohort Study. J. Crohn’s Colitis.

[33] Ding Z., Sherlock M., Chan A.K.C., Zachos M. (2022). Venous Thromboembolism in Pediatric Inflammatory Bowel Disease: An 11-Year Population-Based Nested Case-Control Study in Canada. Blood Coagul. Fibrinolysis.

[34] Witmer C.M., Takemoto C.M. (2017). Pediatric Hospital Acquired Venous Thromboembolism. Front. Pediatr..

[35] Arlikar S.J., Atchison C.M., Amankwah E.K., Ayala I.A., Barrett L.A., Branchford B.R., Streiff M.B., Takemoto C.M., Goldenberg N.A. (2015). Development of a New Risk Score for Hospital-Associated Venous Thromboembolism in Critically Ill Children Not Undergoing Cardiothoracic Surgery. Thromb. Res..

[36] Zaidi A.U., Hutchins K.K., Rajpurkar M. (2017). Pulmonary Embolism in Children. Front. Pediatr..

[37] Agha B.S., Sturm J.J., Simon H.K., Hirsh D.A. (2013). Pulmonary Embolism in the Pediatric Emergency Department. Pediatrics.

[38] Pelland-Marcotte M.-C., Tucker C., Klaassen A., Avila M.L., Amid A., Amiri N., Williams S., Halton J., Brandão L.R. (2019). Outcomes and Risk Factors of Massive and Submassive Pulmonary Embolism in Children: A Retrospective Cohort Study. Lancet Haematol..

[39] Hancock H.S., Wang M., Gist K.M., Gibson E., Miyamoto S.D., Mourani P.M., Manco-Johnson M.J., Goldenberg N.A. (2013). Cardiac Findings and Long-Term Thromboembolic Outcomes Following Pulmonary Embolism in Children: A Combined Retrospective-Prospective Inception Cohort Study. Cardiol. Young.

[40] Brandão L.R., Simpson E.A., Lau K.K. (2011). Neonatal Renal Vein Thrombosis. Semin. Fetal Neonatal Med..

[41] Williams S., Chan A.K. (2011). Neonatal Portal Vein Thrombosis: Diagnosis and Management. Semin. Fetal Neonatal Med..

[42] Tang C.X., Schoepf U.J., Chowdhury S.M., Fox M.A., Zhang L.J., Lu G.M. (2015). Multidetector Computed Tomography Pulmonary Angiography in Childhood Acute Pulmonary Embolism. Pediatr. Radiol..

[43] Degerstedt S.G., Winant A.J., Lee E.Y. (2022). Pediatric Pulmonary Embolism: Imaging Guidelines and Recommendations. Radiol. Clin..

[44] Monagle P., Azzam M., Bercovitz R., Betensky M., Bhat R., Biss T., Branchford B., Brandão L.R., Chan A.K.C., Faustino E.V.S. (2025). American Society of Hematology/International Society on Thrombosis and Haemostasis 2024 Updated Guidelines for Treatment of Venous Thromboembolism in Pediatric Patients. Blood Adv..

[45] Goldenberg N.A., Kittelson J.M., Abshire T.C., Bonaca M., Casella J.F., Dale R.A., Halperin J.L., Hamblin F., Kessler C.M., Manco-Johnson M.J. (2022). Effect of Anticoagulant Therapy for 6 Weeks vs. 3 Months on Recurrence and Bleeding Events in Patients Younger Than 21 Years of Age with Provoked Venous Thromboembolism: The Kids-DOTT Randomized Clinical Trial. JAMA.

[46] Male C., Lensing A.W.A., Palumbo J.S., Kumar R., Nurmeev I., Hege K., Bonnet D., Connor P., Hooimeijer H.L., Torres M. (2020). Rivaroxaban Compared with Standard Anticoagulants for the Treatment of Acute Venous Thromboembolism in Children: A Randomised, Controlled, Phase 3 Trial. Lancet Haematol..

[47] Halton J., Brandão L.R., Luciani M., Bomgaars L., Chalmers E., Mitchell L.G., Nurmeev I., Sharathkumar A., Svirin P., Gorbatikov K. (2021). Dabigatran Etexilate for the Treatment of Acute Venous Thromboembolism in Children (DIVERSITY): A Randomised, Controlled, Open-Label, Phase 2b/3, Non-Inferiority Trial. Lancet Haematol..

[48] Palumbo J.S., Lensing A.W.A., Brandão L.R., Hooimeijer H.L., Kenet G., van Ommen H., Pap A.F., Majumder M., Kubitza D., Thelen K. (2022). Anticoagulation in Pediatric Cancer-Associated Venous Thromboembolism: A Subgroup Analysis of EINSTEIN-Jr. Blood Adv..

[49] Klaassen I.L.M., Sol J.J., Suijker M.H., Fijnvandraat K., van de Wetering M.D., van Ommen C.H. (2019). Are Low-Molecular-Weight Heparins Safe and Effective in Children? A Systematic Review. Blood Rev..

[50] Schloemer N.J., Abu-Sultaneh S., Hanson S.J., Yan K., Hoffmann R.G., Punzalan R.C., Havens P.L. (2014). Higher Doses of Low-Molecular-Weight Heparin (Enoxaparin) Are Needed to Achieve Target Anti-Xa Concentrations in Critically Ill Children. Pediatr. Crit. Care Med..

[51] Schechter T., Finkelstein Y., Ali M., Kahr W.H., Williams S., Chan A.K., Deveber G., Brandão L.R. (2012). Unfractionated Heparin Dosing in Young Infants: Clinical Outcomes in a Cohort Monitored with Anti-Factor Xa Levels. J. Thromb. Haemost..

[52] Hanslik A., Kitzmüller E., Tran U.S., Thom K., Karapetian H., Prutsch N., Voitl J., Michel-Behnke I., Newall F., Male C. (2015). Monitoring Unfractionated Heparin in Children: A Parallel-Cohort Randomized Controlled Trial Comparing 2 Dose Protocols. Blood.

[53] Brandão L.R., Albisetti M., Halton J., Bomgaars L., Chalmers E., Mitchell L.G., Nurmeev I., Svirin P., Kuhn T., Zapletal O. (2020). Safety of Dabigatran Etexilate for the Secondary Prevention of Venous Thromboembolism in Children. Blood.

[54] Kiskaddon A.L., Branstetter J., Williams P., Ignjatovic V., Memken A., Wilhoit K., Goldenberg N.A. (2025). Intravenous Direct Thrombin Inhibitors for Acute Venous Thromboembolism or Heparin-Induced Thrombocytopenia with Thrombosis in Children: A Systematic Review of the Literature. Semin. Thromb. Hemost..

[55] Tarango C., Manco-Johnson M.J. (2017). Pediatric Thrombolysis: A Practical Approach. Front. Pediatr..

[56] Pedersen L.H., Villadsen G.B., Hellfritzsch M., Hvas A.-M. (2022). Prophylaxis of Venous Thromboembolism in Children: A Systematic Review. Semin. Thromb. Hemost..

[57] Branchford B.R., Betensky M., Goldenberg N.A. (2018). Pediatric Issues in Thrombosis and Hemostasis: The How and Why of Venous Thromboembolism Risk Stratification in Hospitalized Children. Thromb. Res..

[58] Walker S.C., Creech C.B., Domenico H.J., French B., Byrne D.W., Wheeler A.P. (2021). A Real-Time Risk-Prediction Model for Pediatric Venous Thromboembolic Events. Pediatrics.

[59] Faustino E.V.S., Shabanova V., Raffini L.J., Kandil S.B., Li S., Pinto M.G., Cholette J.M., Hanson S.J., Nellis M.E., Silva C.T. (2021). Efficacy of Early Prophylaxis Against Catheter-Associated Thrombosis in Critically Ill Children: A Bayesian Phase 2b Randomized Clinical Trial. Crit. Care Med..

[60] Tullius B.P., Athale U., van Ommen C., Chan A.K.C., Palumbo J.S., Balagtas J.M.S. (2018). The Identification of At-Risk Patients and Prevention of Venous Thromboembolism in Pediatric Cancer: Guidance from the SSC of the ISTH. J. Thromb. Haemost..

[61] O’Brien S.H., Rodriguez V., Lew G., Newburger J.W., Schultz C.L., Orgel E., Derr K., Ranalli M.A., Esbenshade A.J., Hochberg J. (2024). Apixaban versus No Anticoagulation for the Prevention of Venous Thromboembolism in Children with Newly Diagnosed Acute Lymphoblastic Leukaemia or Lymphoma (PREVAPIX-ALL): A Phase 3, Open-Label, Randomised, Controlled Trial. Lancet Haematol..

[62] Greiner J., Schrappe M., Claviez A., Zimmermann M., Niemeyer C., Kolb R., Eberl W., Berthold F., Bergsträsser E., Gnekow A. (2019). THROMBOTECT—A Randomized Study Comparing low-molecular-weight heparin, Antithrombin and Unfractionated Heparin for Thromboprophylaxis during Induction Therapy of Acute Lymphoblastic Leukemia in Children and Adolescents. Haematologica.

[63] Sibson K.R., Biss T.T., Furness C.L., Grainger J.D., Hough R.E., Macartney C., Payne J.H., Chalmers E.A., Haematology T.B.S.F. (2018). BSH Guideline: Management of Thrombotic and Haemostatic Issues in Paediatric Malignancy. Br. J. Haematol..

[64] Giordano P., Saracco P., Grassi M., Luciani M., Banov L., Carraro F., Crocoli A., Cesaro S., Zanazzo G.A., Molinari A.C. (2015). Recommendations for the Use of Long-Term Central Venous Catheter (CVC) in Children with Hemato-Oncological Disorders: Management of CVC-Related Occlusion and CVC-Related Thrombosis (AIEOP). Ann. Hematol..

[65] Mahajerin A., Petty J.K., Hanson S.J., Thompson A.J., O’Brien S.H., Streck C.J., Petrillo T.M., Faustino E.V.S. (2017). Prophylaxis against venous thromboembolism in pediatric trauma: A practice management guideline from the Eastern Association for the Surgery of Trauma and the Pediatric Trauma Society. J. Trauma Acute Care Surg..

[66] Hanson S.J., Faustino E.V.S., Mahajerin A., O’Brien S.H., Streck C.J., Thompson A.J., Petrillo T.M., Petty J.K. (2016). Recommendations for venous thromboembolism prophylaxis in pediatric trauma patients: A national, multidisciplinary consensus study. J. Trauma Acute Care Surg..

[67] Vegting I.L., Tabbers M.M., Benninga M.A., Wilde J.C., Serlie M.J., Tas T.A., Jonkers C.F., van Ommen C.H. (2012). Prophylactic Anticoagulation Decreases Catheter-Related Thrombosis and Occlusion in Children with Home Parenteral Nutrition. JPEN J. Parenter. Enter. Nutr..

[68] McCrindle B.W., Michelson A.D., Van Bergen A.H., Horowitz E.S., Sandoval J.P., Justino H., Harris K.C., Jefferies J.L., Pina L.M., Peluso C. (2021). Thromboprophylaxis for Children Post-Fontan Procedure: Insights from the UNIVERSE Study. J. Am. Heart Assoc..

[69] Clark H.H., Ballester L., Whitworth H., Raffini L., Witmer C. (2022). Prevention of Recurrent Thrombotic Events in Children with Central Venous Catheter-Associated Venous Thrombosis. Blood.

[70] Avila L., Betensky M., Cohen C., Ahuja S., Goldenberg N., Zia A. (2024). Clinical Care of Pediatric Patients with or at Risk of Post-Thrombotic Syndrome: Guidance from the ISTH SSC Subcommittee on Pediatric and Neonatal Thrombosis and Hemostasis. J. Thromb. Haemost..

[71] Betensky M., Mosha M., Ashour D., Bruzek S., Amankwah E.K., Campbell C.M., Frye W., Kahn S.R., Ignjatovic V., Goldenberg N.A. (2026). Post-Thrombotic Syndrome-Related Chronic Limb Pain in Children: Findings from the Kids-DOTT Trial. J. Thromb. Haemost..

[72] Bastas D., Brandão L.R., Vincelli J., Wilson D., Perrem L., Guerra V., Wong G., Bentley R.F., Tole S., Schneiderman J.E. (2024). Long-Term Outcomes of Pulmonary Embolism in Children and Adolescents. Blood.

[73] Ignjatovic V., Mertyn E., Monagle P. (2011). The Coagulation System in Children: Developmental and Pathophysiological Considerations. Semin. Thromb. Hemost..

[74] El-Naggar W., Yoon E.W., McMillan D., Afifi J., Mitra S., Singh B., da Silva O., Lee S.K., Shah P.S. (2020). Epidemiology of Thrombosis in Canadian Neonatal Intensive Care Units. J. Perinatol..

[75] Bhat R., Kumar R., Kwon S., Murthy K., Liem R.I. (2018). Risk Factors for Neonatal Venous and Arterial Thromboembolism in the Neonatal Intensive Care Unit—A Case Control Study. J. Pediatr..

[76] Haddad H., Lee K.-S., Higgins A., McMillan D., Price V., El-Naggar W. (2014). Routine Surveillance Ultrasound for the Management of Central Venous Catheters in Neonates. J. Pediatr..

[77] Rizzi M., Goldenberg N., Bonduel M., Revel-Vilk S., Amankwah E., Albisetti M. (2018). Catheter-Related Arterial Thrombosis in Neonates and Children: A Systematic Review. Thromb. Haemost..

[78] Dubbink-Verheij G.H., Pelsma I.C.M., van Ommen C.H., Smits-Wintjens V.E., Visser R., Steggerda S.J., Pas A.B.T., Lopriore E. (2018). Femoral Vein Catheter Is an Important Risk Factor for Catheter-Related Thrombosis in (Near-)Term Neonates. J. Pediatr. Hematol. Oncol..

[79] Tieu P., Paes B., Ahmed A., Matino D., Chan A., Bhatt M. (2020). Inferior Vena Cava Syndrome in Neonates: An Evidence-Based Systematic Review of the Literature. Pediatr. Blood Cancer.

[80] Bersani I., Piersigilli F., Iacona G., Savarese I., Campi F., Dotta A., Auriti C., Di Stasio E., Garcovich M. (2021). Incidence of Umbilical Vein Catheter-Associated Thrombosis of the Portal System: A Systematic Review and Meta-Analysis. World J. Hepatol..

[81] Bhatt M.D., Patel V., Butt M.L., Chan A.K., Paes B., The Thrombosis and Hemostasis in Newborns (THiN) Group (2019). Outcomes Following Neonatal Portal Vein Thrombosis: A Descriptive, Single-Center Study and Review of Anticoagulant Therapy. Pediatr. Blood Cancer.

[82] Ouellette A.C., Darling E.K., Sivapathasundaram B., Babe G., Perez R., Chan A.K., Chanchlani R. (2020). Incidence, Risk Factors, and Outcomes of Neonatal Renal Vein Thrombosis in Ontario: Population-Based Cohort Study. Kidney360.

[83] Kraft J.K., Brandão L.R., Navarro O.M. (2011). Sonography of Renal Venous Thrombosis in Neonates and Infants: Can We Predict Outcome?. Pediatr. Radiol..

[84] Gibson K., Sharp R., Ullman A., Morris S., Kleidon T., Esterman A. (2021). Adverse Events Associated with Umbilical Catheters: A Systematic Review and Meta-Analysis. J. Perinatol..

[85] Price V.E., Ledingham D.L., Krümpel A., Chan A.K. (2011). Diagnosis and Management of Neonatal Purpura Fulminans. Semin. Fetal Neonatal Med..

[86] Limperger V., Klostermeier U.C., Kenet G., Holzhauer S., Gelas M.A., Finckh U., Junker R., Heller C., Zieger B., Kurnik K. (2014). Clinical and Laboratory Characteristics of Children with Venous Thromboembolism and Protein C-Deficiency: An Observational Israeli-German Cohort Study. Br. J. Haematol..

[87] Jones S., Monagle P., Newall F. (2020). Do Asymptomatic Clots in Children Matter?. Thromb. Res..

[88] Yang J.Y., Williams S., Brandão L.R., Chan A.K. (2010). Neonatal and Childhood Right Atrial Thrombosis: Recognition and a Risk-Stratified Treatment Approach. Blood Coagul. Fibrinolysis.

[89] Yang J.Y., Williams S., Brandão L.R., Chan A.K., Mondal T. (2013). Neonatal and Childhood Right Atrial Thrombosis: Critical Clot Size. Blood Coagul. Fibrinolysis.

[90] Ting J., Yeung K., Paes B., Chan A.K., Petropoulos J.-A., Banfield L., Bhatt M.D. (2021). How to Use Low-Molecular-Weight Heparin to Treat Neonatal Thrombosis in Clinical Practice. Blood Coagul. Fibrinolysis.

[91] Bhatt M.D., Paes B.A., Chan A.K. (2016). How to Use Unfractionated Heparin to Treat Neonatal Thrombosis in Clinical Practice. Blood Coagul. Fibrinolysis.

[92] Diaz R., Moffett B.S., Karabinas S., Guffrey D., Mahoney D.H., Yee D.L. (2015). Antithrombin Concentrate Use in Children Receiving Unfractionated Heparin for Acute Thrombosis. J. Pediatr..

[93] Young G., Boshkov L.K., Sullivan J., Raffini L., Cox D., Boyle D., Kallender H., Tarka E., Soffer J., Hursting M. (2011). Argatroban Therapy in Pediatric Patients Requiring Nonheparin Anticoagulation: An Open-Label, Safety, Efficacy, and Pharmacokinetic Study. Pediatr. Blood Cancer.

[94] El-Segaier M., Khan M.A., Khan Z.U., Momenah T., Galal M.O. (2015). Recombinant Tissue Plasminogen Activator in the Treatment of Neonates with Intracardiac and Great Vessels Thrombosis. Pediatr. Cardiol..

[95] Grizante-Lopes P., Garanito M.P., Celeste D.M., Krebs V.L.J., Carneiro J.D.A. (2020). Thrombolytic Therapy in Preterm Infants: Fifteen-Year Experience. Pediatr. Blood Cancer.

[96] de Kort E.H., Vrancken S.L., van Heijst A.F., Binkhorst M., Cuppen M.P., Brons P.P. (2011). Long-Term Subcutaneous Protein C Replacement in Neonatal Severe Protein C Deficiency. Pediatrics.

[97] Lau K.K., Chan H.H., Massicotte P., Chan A.K. (2014). Thrombotic Complications of Neonates and Children with Congenital Nephrotic Syndrome. Curr. Pediatr. Rev..

[98] Pelland-Marcotte M.-C., Pole J.D., Kulkarni K., Athale U., Stammers D., Sabapathy C., Halparin J., Brandão L.R., Sung L. (2018). Thromboembolism Incidence and Risk Factors in Children with Cancer: A Population-Based Cohort Study. Thromb. Haemost..

[99] O’Brien S.H., Klima J., Termuhlen A.M., Kelleher K.J. (2011). Venous Thromboembolism and Adolescent and Young Adult Oncology Inpatients in US Children’s Hospitals, 2001 to 2008. J. Pediatr..

[100] Spavor M., Halton J., Dietrich K., Israels S., Shereck E., Yong J., Yasui Y., Mitchell L.G. (2016). Age at Cancer Diagnosis, Non-O Blood Group and Asparaginase Therapy Are Independently Associated with deep vein thrombosis in Pediatric Oncology Patients: A Risk Model. Thromb. Res..

[101] Prasca S., Carmona R., Ji L., Ko R.H., Bhojwani D., Rawlins Y.A., Mittelman S.D., Young G., Orgel E. (2018). Obesity and Risk for Venous Thromboembolism from Contemporary Therapy for Pediatric Acute Lymphoblastic Leukemia. Thromb. Res..

[102] Tuckuviene R., Ranta S., Albertsen B.K., Andersson N.G., Bendtsen M.D., Frisk T., Gunnes M.W., Helgestad J., Heyman M.M., Jonsson O.G. (2016). Prospective Study of Thromboembolism in 1038 Children with Acute Lymphoblastic Leukemia: A Nordic Society of Pediatric Hematology and Oncology (NOPHO) Study. J. Thromb. Haemost..

[103] Olivi M., Di Biase F., Lanzarone G., Arrigo G., Martella F., Apolito V., Secreto C., Freilone R., Bruno B., Audisio E. (2023). Thrombosis in Acute Myeloid Leukemia: Pathogenesis, Risk Factors and Therapeutic Challenges. Curr. Treat. Options Oncol..

[104] Gartrell J., Kaste S.C., Sandlund J.T., Flerlage J., Zhou Y., Cheng C., Estepp J., Metzger M.L. (2020). The Association of Mediastinal Mass in the Formation of Thrombi in Pediatric Patients with Non-Lymphoblastic Lymphomas. Pediatr. Blood Cancer.

[105] Albisetti M., Kellenberger C.J., Bergsträsser E., Niggli F., Kroiss S., Rizzi M., Schmugge M. (2013). Port-a-Cath-Related Thrombosis and Postthrombotic Syndrome in Pediatric Oncology Patients. J. Pediatr..

[106] Lyman G.H., Carrier M., Ay C., Di Nisio M., Hicks L.K., Khorana A.A., Leavitt A.D., Lee A.Y.Y., Macbeth F., Morgan R.L. (2021). American Society of Hematology 2021 Guidelines for Management of Venous Thromboembolism: Prevention and Treatment in Patients with Cancer. Blood Adv..

[107] Barg A.A., Levy-Mendelovich S., Gilad O., Yacobovich J., Tamarin I., Budnik I., Golan H., Toren A., Kenet G. (2022). Rivaroxaban Treatment among Children with Cancer-Associated Thromboembolism: Real-World Data. Pediatr. Blood Cancer.

[108] Valenti G.G., Sabo C., Hyde M., Rajpurkar M. (2024). Real-World Experience of Direct Oral Anticoagulant Use in a Single Pediatric Center. Pediatr. Blood Cancer.

[109] Qureshi A., Mitchell C., Richards S., Vora A., Goulden N. (2010). Asparaginase-Related Venous Thrombosis in UKALL 2003—Re-Exposure to Asparaginase Is Feasible and Safe. Br. J. Haematol..

[110] Pelland-Marcotte M.C., Kulkarni K., Athale U.H., Pole J.D., Brandão L.R., Sung L. (2021). Thrombosis Is Associated with Worse Survival in Children with Acute Lymphoblastic Leukemia: A Report from CYP-C. Am. J. Hematol..

[111] Dhariwal N., Gollamudi V.R.M., Sangeetha K.P., Parambil B.C., Moulik N.R., Dhamne C., Prasad M., Vora T., Chinnaswamy G., Kembhavi S. (2023). Pediatric Cancer-Associated Thrombosis: Analysis from a Tertiary Care Cancer Center in India. Pediatr. Blood Cancer.

[112] Mitchell L., Lambers M., Flege S., Kenet G., Li-Thiao-Te V., Holzhauer S., Bidlingmaier C., Frühwald M.C., Heller C., Schmidt W. (2010). Validation of a Predictive Model for Identifying an Increased Risk for Thromboembolism in Children with Acute Lymphoblastic Leukemia: Results of a Multicenter Cohort Study. Blood.

[113] Pelland-Marcotte M.-C., Tole S., Pechlivanoglou P., Brandão L.R. (2019). Effectiveness and Safety of Primary Thromboprophylaxis in Children with Cancer: A Systematic Review of the Literature and Network Meta-Analysis. Thromb. Haemost..

[114] Otite F.O., Vanguru H., Anikpezie N., Patel S.D., Chaturvedi S. (2022). Contemporary Incidence and Burden of Cerebral Venous Sinus Thrombosis in Children of the United States. Stroke.

[115] Ichord R. (2017). Cerebral Sinovenous Thrombosis. Front. Pediatr..

[116] Ferriero D.M., Fullerton H.J., Bernard T.J., Billinghurst L., Daniels S.R., DeBaun M.R., deVeber G., Ichord R.N., Jordan L.C., Massicotte P. (2019). Management of Stroke in Neonates and Children: A Scientific Statement from the American Heart Association/American Stroke Association. Stroke.

[117] Hedlund G.L. (2013). Cerebral Sinovenous Thrombosis in Pediatric Practice. Pediatr. Radiol..

[118] Moharir M.D., Shroff M., Stephens D., Pontigon A.M., Chan A., MacGregor D., Mikulis D., Adams M., deVeber G. (2010). Anticoagulants in Pediatric Cerebral Sinovenous Thrombosis: A Safety and Outcome Study. Ann. Neurol..

[119] Connor P., Sánchez van Kammen M., Lensing A.W.A., Chalmers E., Kálláy K., Hege K., Simioni P., Biss T., Bajolle F., Bonnet D. (2020). Safety and Efficacy of Rivaroxaban in Pediatric Cerebral Venous Thrombosis (EINSTEIN-Jr CVT). Blood Adv..

[120] Ichord R.N., Benedict S.L., Chan A.K.C., Kirkham F.J., Nowak-Göttl U., International Paediatric Stroke Study Group (2015). Paediatric Cerebral Sinovenous Thrombosis: Findings of the International Paediatric Stroke Study. Arch. Dis. Child..

[121] Moharir M.D., Shroff M., Pontigon A.M., Maclusky I., Macgregor D., Kirton A., deVeber G. (2011). A Prospective Outcome Study of Neonatal Cerebral Sinovenous Thrombosis. J. Child Neurol..

[122] Connelly C.R., Laird A., Barton J.S., Fischer P.E., Krishnaswami S., Schreiber M.A., Zonies D.H., Watters J.M. (2016). A clinical tool for the prediction of venous thromboembolism in pediatric trauma patients. JAMA Surg..

[123] Yen J., Van Arendonk K.J., Streiff M.B., McNamara L., Stewart F.D., Conner K.G.R., Thompson R.E., Haut E.R., Takemoto C.M. (2016). Risk factors for venous thromboembolism in pediatric trauma patients and validation of a novel scoring system: The risk of clots in kids with trauma score. Pediatr. Crit. Care Med..

[124] Goldenberg N.A., Sochet A., Albisetti M., Biss T., Bonduel M., Jaffray J., MacLaren G., Monagle P., O’Brien S., Raffini L. (2020). Consensus-based clinical recommendations and research priorities for anticoagulant thromboprophylaxis in children hospitalized for COVID-19-related illness. J. Thromb. Haemost..

[125] Huang J.Y., Monagle P., Massicotte M.P., VanderPluym C.J. (2018). Antithrombotic therapies in children on durable ventricular assist devices: A literature review. Thromb. Res..

[126] Rychik J., Atz A.M., Celermajer D.S., Deal B.J., Gatzoulis M.A., Gewillig M.H., Hsia T.-Y., Hsu D.T., Kovacs A.H., McCrindle B.W. (2019). Evaluation and management of the child and adult with Fontan circulation: A scientific statement from the American Heart Association. Circulation.

[127] Groot N., de Graeff N., Avcin T., Bader-Meunier B., Dolezalova P., Feldman B., Kenet G., Koné-Paut I., Lahdenne P., Marks S.D. (2017). European evidence-based recommendations for diagnosis and treatment of paediatric antiphospholipid syndrome: The SHARE initiative. Ann. Rheum. Dis..

[128] Avila L., Amiri N., De R., Vincelli J., Pullenayegum E., Brandão L.R. (2021). Compression garments for the management of pediatric post-thrombotic syndrome: A prospective longitudinal study. J. Thromb. Haemost..

[129] Kumar R., Stanek J., Creary S., Dunn A., O’Brien S.H. (2018). Prevalence and risk factors for venous thromboembolism in children with sickle cell disease: An administrative database study. Blood Adv..

[130] Rangarajan H.G., Stanek J.R., Abu-Arja R., Bajwa R.P., Auletta J.J., Lee D.A., O’Brien S.H., Kumar R. (2018). Venous thromboembolism in pediatric hematopoietic cell transplant: A multicenter cohort study. Biol. Blood Marrow Transpl..

